# USP18 and ISG15 coordinately impact on SKP2 and cell cycle progression

**DOI:** 10.1038/s41598-019-39343-7

**Published:** 2019-03-11

**Authors:** Françoise Vuillier, Zhi Li, Pierre-Henri Commere, Lasse Toftdal Dynesen, Sandra Pellegrini

**Affiliations:** 1 Institut Pasteur, Unit of Cytokine Signaling, Inserm U1221, 75724 Paris, France; 2 Institut Pasteur, Flow Cytometry Platform, 75724 Paris, France

## Abstract

USP18 is an isopeptidase that cleaves the ubiquitin-like ISG15 from conjugates and is also an essential negative feedback regulator of type I interferon signaling. We and others reported that USP18 protein is stabilized by ISG15 and targeted for degradation by SKP2 (S-phase kinase associated protein 2), the substrate-recognition subunit of the SCF^SKP2^ ubiquitin E3 ligase complex, which operates in cell cycle progression. Here, we have analyzed how, under non stimulated conditions, USP18, ISG15 and SKP2 communicate with each other, by enforcing or silencing their expression. We found that USP18 and SKP2 interact and that free ISG15 abrogates the complex, liberating USP18 from degradation and concomitantly driving SKP2 to degradation and/or ISGylation. These data reveal a dynamic interplay where the substrate USP18 stabilizes SKP2, both exogenous and endogenous. Consistent with this we show that silencing of baseline USP18 slows down progression of HeLa S3 cells towards S phase. Our findings point to USP18 and ISG15 as unexpected new SKP2 regulators, which aid in cell cycle progression at homeostasis.

## Introduction

ISG15 (IFN-stimulated gene 15) protein is composed of two ubiquitin-like domains separated by a short hinge. The protein is translated as a precursor that is cleaved at the C-ter to yield the mature 15 kDa protein. Monomeric ISG15 is found either free or covalently bound to proteins through ISGylation, a conjugation process that uses an activating E1 enzyme (UBE1L), a conjugating E2 enzyme (UBCH8), and one of three E3 ligases (HERC5, EFP and HHARI) (reviewed in Zhang *et al*.^1^). These enzymes and ISG15 itself are strongly transcriptionally up-regulated by virus-induced type I interferons (IFN-I). Consequently numerous viral and cellular proteins can be modified by ISGylation and thereby contribute to the virus-host interactions and the innate response to infection. Yet, ISGylation is not a prerequisite for ISG15 function, since unconjugated (free) ISG15 has been found to modify a number of viral and host proteins by non covalent binding^2^. Moreover, ISG15 and the ISGylation machinery can be induced in contexts beyond infection and target proteins involved in proliferation, chromatin remodeling, autophagy, cell cycle regulation and innate immune cells activation^2^. Recent studies indicate that, like other post-translational modifications, ISGylation contributes to cellular responses to genotoxic stress, thereby assisting cells to rapidly adapt to dangerous perturbations^3^.

Importantly, ISGylation is transient and reversed by the action of USP18 (ubiquitin-like specific protease 18) (also known as UBP43), the isopeptidase that cleaves ISG15 from conjugates. USP18 is transcriptionally induced by IFN-I and restrains global ISGylation by de-ISGylating substrates and also by inhibiting IFN-I signaling. The function of USP18 as essential negative feedback regulator of IFN-I was first described in *Usp18−/−* mouse models and human cell lines. The identification of rare USP18-deficient patients presenting with severe brain pathology - a novel interferonopathy - confirmed the role of USP18 in restraining IFN signaling^4–7^. Interestingly, ISG15 is also part of this negative feedback loop. Rare *ISG15*-deficient patients present a high level of IFN-stimulated gene transcripts (*ISGs*) in blood cells and develop a mild interferonopathy^8^. Cells from these patients are hyper-responsive to IFN-I and this was ascribed to insufficient levels of USP18. Subsequent studies showed that in humans, but not in mice, ISG15 protects USP18 from proteolysis^9^.

SKP2 (S-phase kinase associated protein 2) was found to target USP18 to ubiquitination and proteosomal degradation^10^. SKP2 is a well-known F-box protein and the substrate-binding subunit of the SCF^SKP2^ ubiquitin E3 ligase complex (SKP1, CUL1, RBX1, SKP2). By targeting to degradation several cell cycle negative regulators, such as p21, p27 and p130, the SCF^SKP2^ complex promotes entry of cells into S phase^11,12^. When SKP2 is ubiquitinated and degraded *via* the APC/C^CDH1^ complex, p27 and p21 are stabilized and promote G1/S phase arrest^13,14^. SKP2 protein level is low in G0/G1 and gradually increases as cells move to S phase to persist until mitosis^11^. SKP2 function is regulated by ubiquitination and other mechanisms that most often influence protein turnover. For instance, phosphorylation of Ser64 and Thr417 protects SKP2 from degradation^15,16^ and acetylation by p300 promotes SKP2 stability and dimerization^17^. Given its key role in regulating the abundance of cell cycle inhibitors, SKP2 is involved in numerous cell cycle-related processes that ultimately influence cell survival, differentiation, apoptosis and many other physiological features. SKP2 is upregulated in many cancers and has a recognized oncogenic potential, often correlating with poor prognosis^12,18–20^.

As summarized above, SKP2 can target USP18 for degradation, while ISG15 has an opposite action, being required for accumulation of USP18 as seen in IFN-stimulated cells. These findings raise questions about the mode of action of ISG15, the interplay of ISG15 and SKP2 and the potential participation of USP18 and ISG15 to cell cycle regulatory processes under homeostatic conditions and/or IFN-stimulated conditions. To address these issues, we aimed to biochemically define how the components of this trio (USP18, ISG15, SKP2) communicate with each other. We also investigated whether baseline USP18, through connecting with SKP2, is implicated in cell cycle progression. Our data reveal a dynamic physical and functional interplay between the three proteins. The involvement of USP18 and ISG15, two IFN-inducible proteins, in global ISGylation, IFN signaling and cell cycle progression point to an intimate relation between homeostatic IFN action and cell fate.

## Results

### ISG15 interferes with the USP18-SKP2 complex

**Figure 1:**
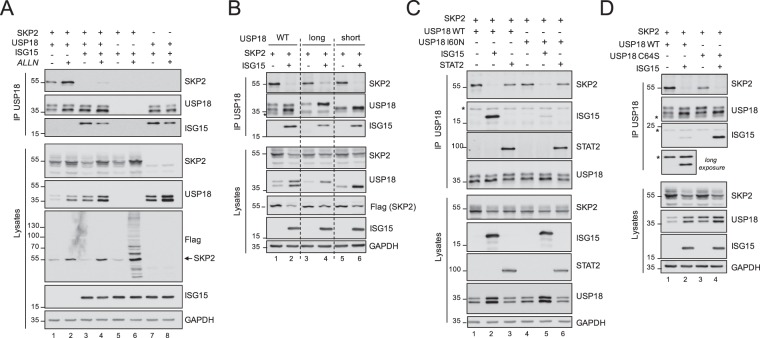
ISG15 abrogates the USP18-SKP2 complex independently of binding to USP18. (**A**) 293T cells were co-transfected with Flag-SKP2, USP18-V5 and/or 3xFlag-ISG15 as indicated. ALLN (50 μM) was added 16 h before cell lysis. Anti-V5 immunoprecipitates (top) and total lysates (bottom) were subject to immunoblot with SKP2 Abs, V5 Abs to reveal USP18 and Flag Abs to reveal ISG15 and SKP2. (**B**) 293T cells were co-transfected with plasmids expressing Flag-SKP2, USP18-V5 (WT, long or short isoforms) with or without 3xFlag-ISG15, as indicated, and processed as described in (**A**), but without addition of ALLN. Asterisk, non specific band. (**C**) Cells were transfected with Flag-SKP2, USP18-V5 WT or the I60N mutant, with and without 3xFlag-ISG15 or STAT2 as indicated, and processed as in (**B**). Asterisk, non specific band. (**D**) Cells were co-transfected with Flag-SKP2, USP18-V5 WT or the C64S mutant with or without 3xFlag ISG15 and processed as in (**B**). Asterisks, non specific bands.

We and others have shown that USP18 and SKP2 are able to interact^8,10^. In a first set of experiments, we analyzed the impact of ISG15 on the USP18-SKP2 complex. For this, 293T cells were transfected with tagged versions of USP18 and SKP2 in the absence or presence of ISG15. By co-immunoprecipitation we confirmed the binding of exogenous SKP2 (55 kDa) to USP18 (Fig. 1A, lane 1). When ISG15 was co-expressed, no such complex was detected and instead ISG15 was found to bind USP18 (lane 3). Since we noticed that the total amount of SKP2 in lysates was reduced when cells expressed ISG15, we performed co-immunoprecipitations on cells incubated with ALLN, a proteasome inhibitor. In this condition the USP18-SKP2 complex was more abundant and yet expression of ISG15 abrogated it (lane 2 *vs* lane 4). Similar conclusions were reached when a non conjugatable form of ISG15 (ΔGG) was transfected (Supplementary Fig. S1), indicating that ISG15 abrogates the complex regarless of its conjugation capacity.

USP18 exists as a short and a long isoform differing at the N-terminus. Each USP18 isoform was individually assayed as described above. Figure 1B shows that SKP2 co-immunoprecipitated with both the long and the short USP18 isoform (lanes 3 and 5) in an ISG15-sensitive manner. In addition, both isoforms complexed with ISG15 (lanes 4 and 6).

To get some insight into the mechanism by which ISG15 acts, we tested the possibility that, in condition of high ISG15, USP18 would switch interacting partner from SKP2 to ISG15. For this, we utilized a USP18 point mutant (USP18-I60N) that is unable to co-immunoprecipitate with ISG15 (Fig. 1C). This mutant interacted with SKP2, and ISG15 abrogated the complex. Hence, we inferred that the action of ISG15 does not require binding to USP18. As additional control, we co-transfected STAT2, another USP18 interactor^21^, which did not interfere with the USP18-SKP2 complex (Fig. 1C).

Since USP18 is a de-ISGylase, we reasoned that, in the presence of excess ISG15, active USP18 may be diverted to ISGylated substrates and this would drive its dissociation from SKP2. This possibility could be excluded since a USP18 mutant that is catalytically dead (USP18-C64S) behaved as USP18 WT (Fig. 1D). Overall, these results indicate that free ISG15 interferes with the USP18-SKP2 complex regarless of the catalytic activity of USP18 or its ISG15-binding potential.

**Figure 2:**
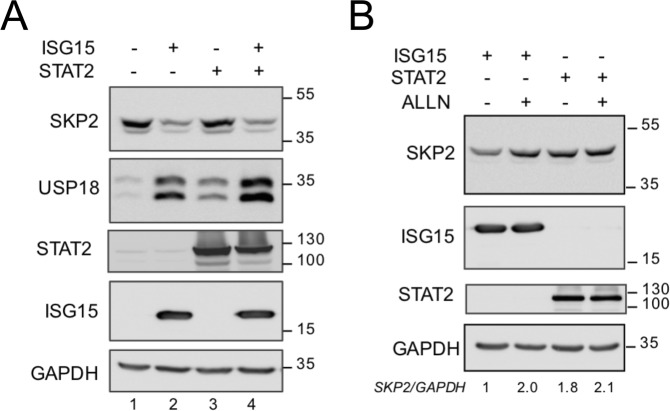
Exogenous ISG15 impacts levels of endogenous USP18 and SKP2. (**A**) 293T cells were transfected with plasmids expressing 3xFlag-ISG15, STAT2 or both as indicated. Lysates were analyzed by western blot as indicated. (**B**) Similar to (**A**), but ALLN (50 μM) was added 16 h before cell lysis. Signals were quantified and reported as SKP2/GAPDH ratios.

We then turned to the study of the endogenous proteins. Our attempts to co-immunoprecipitate endogenous USP18 and SKP2 were not successful, most likely due to low baseline USP18 expression (Fig. 2A, lane 1). Therefore, we asked whether exogenous ISG15 would affect endogenous SKP2 and/or USP18. Interestingly, transfection of ISG15 - but not STAT2 - had a dual effect, in that it lowered SKP2 (47 kDa) and increased USP18 (Fig. 2A). Moreover, when ISG15-transfected cells were incubated with ALLN, SKP2 level was rescued (Fig. 2B), indicating that, in the presence of high ISG15, a fraction of endogenous SKP2 undergoes proteasomal degradation. In line with this, we found that non conjugatable ISG15 (ΔGG) promotes SKP2 ubiquitination (Supplementary Fig. S2).

### SKP2 can be ISGylated

**Figure 3:**
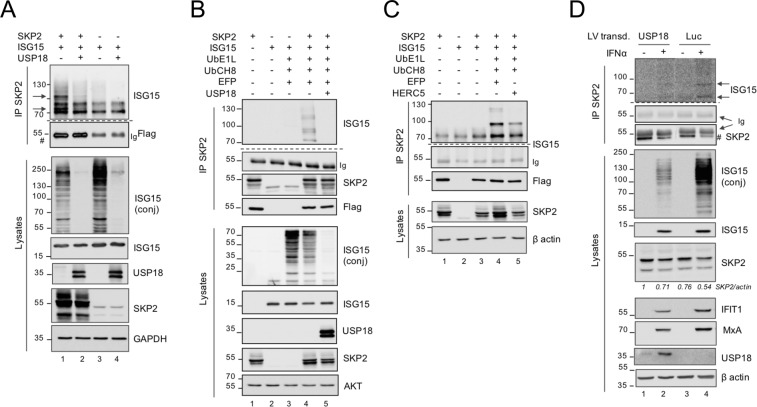
SKP2 is targeted by ISGylation. (**A**) 293T cells were cotransfected with 3xFlag ISG15, Flag-SKP2 and USP18 plasmids, as indicated. ALLN was added 16 h before cell lysis. Lysates were immunoprecipitated with antibodies to SKP2. Immunoprecipitates (IP) and whole cell lysates were immunoblotted as indicated. Arrows, ISGylated SKP2 forms. In lanes 1 and 2 exogenous SKP2 (#) and immunoglobulin heavy chain (Ig) co-migrated at around 55 kDa. (**B**) 293T cells were transfected with plasmids expressing Flag-SKP2, MRGS-6His-ISG15, the ISGylation enzymes (UBE1L, UBCH8, EFP) and USP18 as indicated. Lysates were immunoprecipitated with antibodies to SKP2. Immunoprecipitates (IP) and whole cell lysates were immunoblotted as indicated. The IP samples were blotted first with anti-ISG15 Abs. The membrane was then cut above the 55 kDa marker (Ig) (dotted line) and ISGylated forms of SKP2 were revealed in the upper part using a sensitive ECL reagent and long exposure time. (**C**) Similar to (B), but either EFP or HERC5 plasmid was co-transfected as E3 enzyme. (**D**) *USP18*-deficient patient fibroblasts, lentivirally transduced with either luciferase (Luc) or USP18, were stimulated by IFNα (250 pM) for 24 h. Lysates were immunoprecipitated with antibodies to SKP2. Immunoprecipitates and whole cell lysates were immunoblotted as indicated. Arrows, ISGylated forms of endogenous SKP2. (#), endogenous 47 kDa SKP2, migrating just below Ig. Bands were quantified and normalized to actin. The ratio for USP18 untreated cells as set to 1.

The experiments described showed a reduction of SKP2 levels, exogenous (55 kDa) or endogenous (47 kDa), in cells expressing high ISG15. This reduction is likely due to degradation as shown by the use of ALLN (see Fig. 1A), but it may also result, at least partly, from post-translational modification such as ISGylation. To test whether SKP2 can be conjugated with ISG15, 293T cells were transfected with ISG15, SKP2 and/or USP18 and lysates were subject to SKP2 immunoprecipitation in order to visualize slow migrating ISGylated SKP2 forms. Global ISGylation was detected in total lysates and it disappeared when USP18 was co-expressed (Fig. 3A). In SKP2 immunoprecipitates, ISGylated forms of endogenous SKP2 could not be detected (Fig. 3A, top panel lane 3). In contrast, two ISG15-reactive bands (around 70 and 100 kDa) were visible when SKP2 was transfected (Fig. 3A, top panel lane 1). A third ISG15-reactive band was detected at around 130 kDa when the ISGylation enzymes (UBE1L, UBCH8 and EFP) were co-expressed (Fig. 3B, top panel, lane 4). Notably, all slow migrating bands disappeared in the presence of USP18 (lanes 4 *vs* 5). We also compared the profile of ISGylated SKP2 forms generated in the presence of the E3 ligases EFP and HERC5. As shown in Fig. 3C, EFP was found to be more efficient than HERC5. Overall, these results demonstrate that, under condition of forced ISGylation, a small fraction of SKP2 can be conjugated to ISG15.

The low amount of ISGylated SKP2 precluded the identification of the lysine residue(s) targeted by ISGylation by mass spectrometry. We attempted to identify major ISG15-acceptor sites by studying lysine to alanine mutant forms of SKP2. All lysine residues were mutated, individually or in groups, and each mutant was tested for expression and ISGylation profile, but no consistent differences were found (Supplementary Fig. S3). This confirms previous studies suggesting a low specificity of lysine residues in the ISGylation process^22,23^.

To reveal ISGylation of endogenous SKP2, we turned to primary dermal fibroblasts derived from a USP18-deficient patient^7^. These cells exhibit a strong and persistent response to IFNα/β and, due to the absence of USP18, they present a high level of global ISGylation. Patient fibroblasts, transduced with control luciferase (Luc) or with USP18, were treated with IFNα and SKP2 was immunoprecipitated. Two weak ISGylated SKP2 forms with the expected molecular weights were detected only in IFNα-treated USP18-deficient cells (Luc) (Fig. 3D, lane 4). These results indicate that a minor fraction of endogenous SKP2 can be targeted by ISGylation.

### IFNβ causes a reduction of SKP2

**Figure 4:**
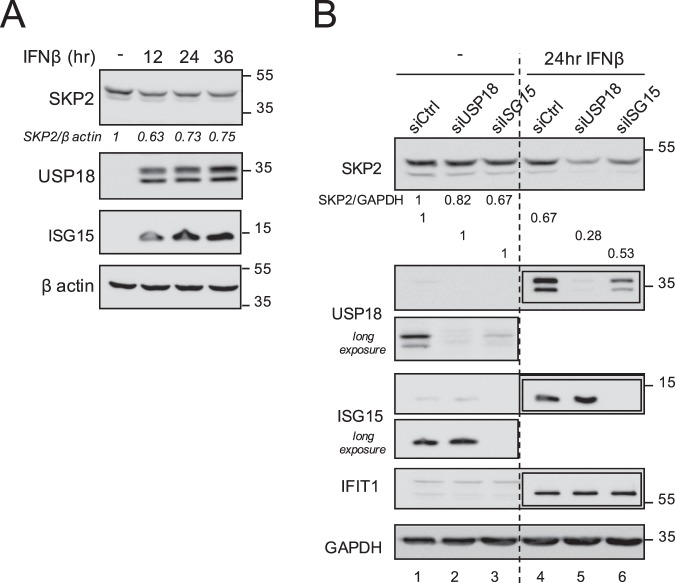
IFNβ impacts endogenous SKP2 in HeLa S3 cells. (**A**) HeLa S3 cells were treated with IFNβ (500 pM) for the time indicated and lysed. Levels of endogenous SKP2 and USP18 were analyzed by western blot of whole cell lysates. (**B**) HeLa S3 cells were transfected with control siRNA (siCTRL) or siRNA targeting USP18 or ISG15. Cells were treated with IFNβ for 24 h and compared with untreated cells. Lysates were analyzed by western blot with anti-SKP2, anti-USP18 or anti-ISG15 antibodies. IFIT1 protein was monitored as control of IFN stimulation. Boxed membranes: 20 x less cell lysate was used. SKP2 band was normalized to GAPDH. Results are reported as the ratio of SKP2 to GAPDH. The ratio obtained for unstimulated cells was set to 1. Long exposure: time extended 3×, after hiding the right portion of the membrane.

We have shown that overexpression of ISG15 leads to a reduction of the level of endogenous SKP2 (Fig. 2). Since type I IFN is a potent inducer of ISG15, we asked whether treatment with this cytokine would affect the level of SKP2 in HeLa S3 cells. Indeed, a reduction of SKP2 (about 30%) was detected as early as 12 h after IFNβ stimulation (Fig. 4A). Since USP18 is also induced by IFN, we tried to dissociate the individual contribution of USP18 and ISG15, using RNA interference. Cells were transfected with si-control, si-USP18 or si-ISG15, left unstimulated or stimulated 24 h with IFNβ and levels of SKP2 were compared. Consistent with the data above, in all conditions IFNβ led to a decrease of SKP2 (Figs 4B and S4A). Of note, in ISG15-silenced cells, USP18 levels (basal and induced) were lower than in control cells (Fig. 4B, lane 1 *vs* 3 and lane 4 *vs* 6), making it difficult to determine the individual contributions. Overall, these data indicate that in IFN-stimulated HeLa S3 cells, the increased USP18 and ISG15 do impact on SKP2 in an interdependent manner.

### USP18 impacts on cell cycle progression

**Figure 5:**
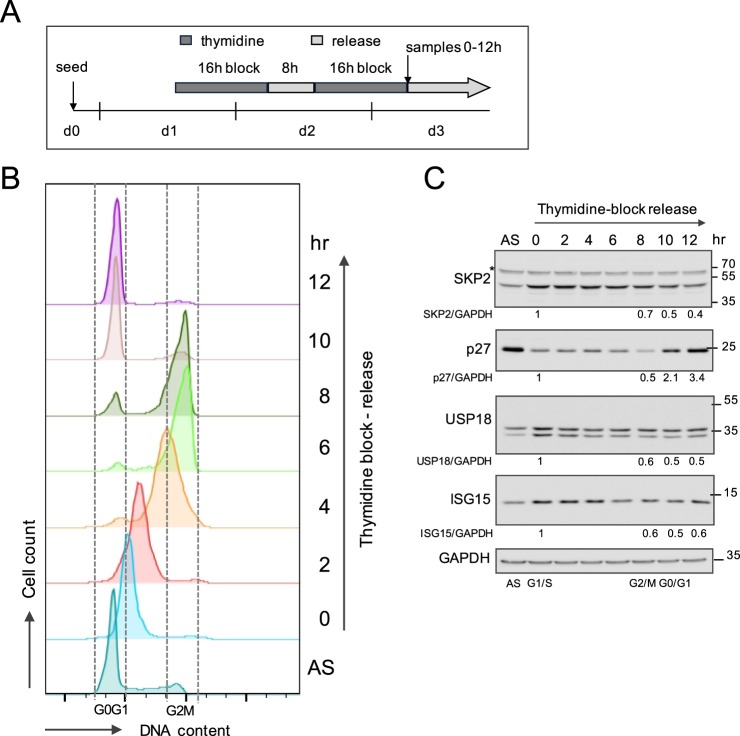
Synchronized HeLa cells are analyzed for SKP2 and ISG proteins. (**A**) Schematics of HeLa S3 cells synchronization in G1/S arrest by double thymidine block and release into fresh medium. After the second release, samples were collected every 2 h during 12 h. (**B**) Cells were stained with propidium iodide for DNA content and the cell cycle profile was determined by flow cytometry. Debris and sub-G1 phase material were excluded. G0/G1 (2 N) and G2/M (4 N) phases are delineated. (**C**) An aliquot of cells from the samples analyzed in (**B**) was lysed for protein analysis. Lysates were immunoblotted, as indicated. Results are reported as the ratio of each protein to GAPDH. The ratio obtained for G1/S phase was set to 1.

Given the key function of SKP2 in cell cycle and its molecular interplay with USP18 and ISG15, we investigated a potential role of USP18 in cell cycle progression. To this end, we first validated a protocol of cell synchronization. HeLa S3 cells were arrested in G1/S phase by a double thymidine block (Fig. 5A). Following release, samples were collected every 2 h over a period of 12 h for parallel cell cycle profiling by flow cytometry (Fig. 5B) and protein analysis (Fig. 5C). As expected, SKP2 (47 kDa) level decreased during progression to G2/M and reached a minimal level in G0/G1, while the level of p27 was highest in G0/G1 (Fig. 5C). USP18 and ISG15 levels were highest at G1/S and decreased after the release, exhibiting an expression profile similar to that of SKP2.

**Figure 6:**
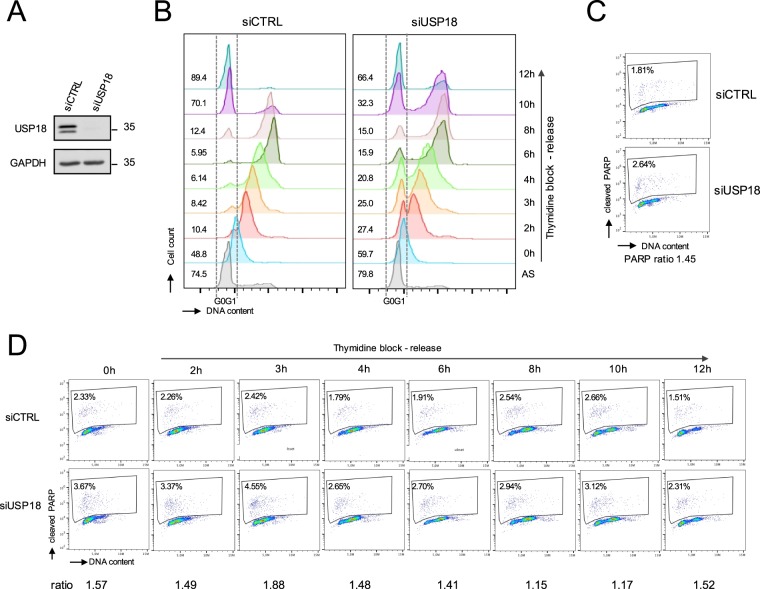
USP18 impacts progression through the cell cycle. (**A**) HeLa S3 cells were retro-transfected with control siRNA (siCTRL) or siRNA targeting USP18 (siUSP18) and left in culture (asynchronous state). Lysates were analyzed by western blot with anti-USP18 antibody. (**B**) Cells were retro-transfected as in (**A**), synchronized in G1/S and released into fresh medium as described in Fig. 5. Cells were stained with propidium iodide for DNA content and the cell cycle profile was determined by flow cytometry. All debris and sub-2N phase were excluded. G0/G1 (2 N) is delineated. Values shown in each histogram represent the percentage of cells in G0/G1 phase. (**C,D**) Similar to (**B**) but cells were fixed, permeabilized and stained with anti-cleaved PARP antibody followed by propidium iodide staining. Cytograms depicted are obtained with asynchronous (**C**) or synchronized (**D**) cells. The value shown in each cytogram represents the percentage of cells positive for cleaved PARP. This percentage was measured in USP18-silenced cells and control cells and a ratio of the values obtained was calculated and defined as PARP ratio as indicated.

To assess whether USP18 had an impact on the progression through the cell cycle, control and USP18-silenced cells were synchronized as described above and analyzed for DNA content. Efficient USP18 silencing in asynchronous cells was confirmed by western blot (Fig. 6A). The cell cycle analysis showed that USP18-silenced cells were well synchronized in G1/S (Fig. 6B). However, 2 to 6 h after the release a substantial fraction of USP18-silenced cells had not yet progressed towards S phase (Fig. 6B). A similar result was obtained with two other USP18 siRNAs (Supplementary Fig. S4B). To test whether cells underwent apoptosis, the experiment was repeated and cells (debris and sub-2N phase excluded) were analyzed by dual staining for DNA content and PARP cleavage. PARP is a characteristic endogenous “death substrate” and its cleavage is considered as hallmark of classical apoptosis. We found that USP18-silenced cells - whether asynchronously proliferating or synchronized - displayed 2 to 3 fold more apoptosis than control cells (Fig. 6C,D), but no further enrichment was measured at any time point after release. Overall, these results indicate that USP18 is a key element for progression through the cell cycle.

## Discussion

Previous observations have suggested the existence of a complex interplay between SKP2, USP18 and ISG15. Here, we have started to disentagle this network with the belief that it could contribute to homeostatic mechanisms of cell proliferation at steady-state and to rapid adaptation to changing environmental conditions. Our main observation is that forced expression of USP18 and ISG15 modulate the level of SKP2, whether endogenous or overexpressed. We found that USP18, substrate of SKP2, can complex with this latter in an interaction which is ISG15-sensitive. Hence, expression of ISG15 abrogates the USP18-SKP2 complex, and this results in destabilization of SKP2 and accumulation of USP18. We also demonstrated that SKP2 can be weakly ISGylated. Furthermore, we showed that HeLa S3 cells that have been acutely depleted of USP18 have an abnormal cell cycle profile, with a fraction of cells retarded in entry into S phase. Taken together, these findings demonstrate for the first time that baseline USP18 and ISG15 impact on the level of SKP2 and cell cycle progression.

SKP2 is a critical substrate-recognition component of the SCF E3 ubiquitin ligase complex. Along with others, we reported that SKP2 interacts with USP18 and promotes its degradative ubiquitination^8,10^. Here, we observed that ISG15 abrogates SKP2 association to USP18 also in the presence of a proteasome inhibitor, indicating a *bona fide* effect of ISG15. Regarding the mechanism, ISG15 may compete out SKP2 by non covalent binding to USP18^8^. However, this is unlikely since ISG15 abrogates a complex of SKP2 and USP18 I60N, this latter unable to bind ISG15. In addition, we cannot explain the dissociation of the complex by ISG15 *via* a diversion of USP18 towards ISGylated substrates, since a catalytically dead mutant (C64S) behaves similarly to USP18 WT. Overall, our results indicate that free ISG15, regardless of its conjugation potential, interferes with the USP18-SKP2 complex through a mechanism that does not require direct binding to USP18 or ISGylation/de-ISGylation events.

The level of SKP2 is regulated by several mechanisms including self-ubiquitination and phosphorylation. Moreover, through reversible modifications of other components of the SCF, like Cul1 neddylation, the SCF^SKP2^ complex is thought to acquire high conformational flexibility that helps to accommodate various substrates^15,16,24,25^. Hence, ISG15 may target SKP2 itself and/or another component of the SCF complex, through covalent or non-covalent binding, thereby inducing a conformational change which prevents access of the substrate USP18 and/or affect the building of the SCF^SKP2^ complex. In this regard, we obtained evidence that SKP2 can be ISGylated and preferentially so by the E3 ligase EFP. Yet, the amount of ISGylated SKP2 is rather small, even in a context where global ISGylation is high as in IFN-stimulated USP18-deficient fibroblasts. Although this is in line with the view that only a small fraction of the total pool of a given protein is ISGylated^26^, we inferred that low levels of SKP2 ISGylation is unlikely to account neither for the disruption of the USP18-SKP2 complex nor for the reduction of SKP2 (47 kDa) observed upon enforced ISG15 expression. Moreover, we confirm that ISG15 promotes USP18 accumulation independently of conjugation^8^. Altogether, these results suggest that a critical determinant of SKP2 reduction is the level of free ISG15 in the cell. For instance, free ISG15 may act, directly or indirectly, on S_64_-P-SKP2, which was shown to be protected from APC^Cdh1^-mediated degradation and to be more stable^15^. In this context, protein ISGylation may indirectly contribute to the maintenance of unconjugated SKP2 by consuming ISG15.

Interestingly, ISG15 not only abrogates the USP18-SKP2 complex, but also renders SKP2 more prone to degradation. We found that, when ISG15 was overexpressed, both exogenous and endogenous SKP2 underwent proteasomal-mediated destruction, condition in which SKP2 self-ubiquitination may occur^24^. USP18-deficient patient fibroblasts rescued with exogenous USP18 exhibited more SKP2 (Fig. 3D, lane 1 *vs* lane 3). Acute silencing of USP18 led to reduced SKP2 level. Altogether these data are highly suggestive of a reciprocal SKP2/USP18 control where USP18, acting as substrate, protects SKP2 from (self) ubiquitination. Such homeostatic regulation has been found for a number of F-box proteins, notably the F-box protein HOS (βTrCP2), whose degradation is attenuated by its substrates β-catenin and the inhibitor of NF-κB, IκB^27,28^. In this scenario, the availability of USP18 in the cell as well as the abundance of the assembled SCF complex will influence SKP2 levels.

**Figure 7:**
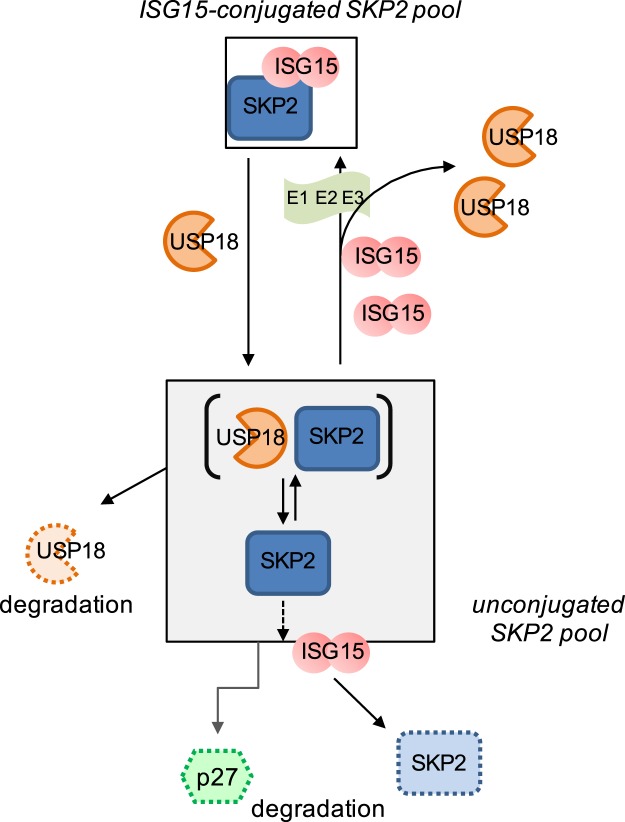
Model of SKP2 regulation by ISG15 and USP18. Two pools of SKP2, a minor ISG15-conjugated one and a more abundant unconjugated one co-exist in a dynamic equilibrium. The unconjugated pool contains the USP18-SKP2 complex (brackets) and SKP2 involved in p27 degradation and cell cycle progression. Changes in the levels of free ISG15 or USP18 will tilt this equilibrium.

In light of our findings, we propose a model where, in baseline condition, USP18 and ISG15 interdependently regulate SKP2 (Fig. 7). In this model, a dynamic exchange exists between unconjugated and ISG15-conjugated SKP2, where the unconjugated pool (47 kDa) comprises SKP2 in complex with USP18 and (active) SKP2 involved in p27 degradation. Any small change in the level of ISG15 and/or USP18 will tilt the equilibrium. In this view, the conjugated SKP2 pool may serve as a reservoir protected from degradation. Any small dysregulation of the ISGylation/deISGylation system is expected to alter the level of (unconjugated) SKP2. Indeed, cells acutely silenced for USP18 or ISG15 displayed lower SKP2 levels (Fig. 4B). Although in USP18-deficient fibroblasts the reduction of SKP2 was rather modest (Fig. 3D) - most likely due to compensatory mechanisms - we cannot rule out that perturbation in SKP2 levels may have contributed to pathogenesis in these patients^7^.

The maintenance of distinct phases during the cell cycle is mainly regulated by phosphorylation and protein degradation reactions involving ubiquitin ligases such as SCF and APC/C complexes^12^. Here we have shown that, in addition to influencing the level of SKP2, baseline USP18 is required for coordinated progression of cells through the G1/S transition, since a subset of USP18-silenced synchronized cells lagged behind. These findings suggest that SKP2 is under the control of USP18 and ISG15 (baseline, stress-induced, virus or IFN-induced), acting as new players in the regulation of cell cycle. At steady state, *ie* in the absence of exogenous IFN or other environmental cues, low baseline ISG15 and USP18 operate towards ensuring a balanced cell cycle progression in HeLa cells. Silencing of USP18 in hepatoma HepG2 cells was shown to cause accumulation of cells in G0/G1 and apoptosis^29^. In other contexts, such as upon viral infection or oxidative stress, ISG15 and USP18 can reach high levels. Although their expression as ISGs is considered as indicator of inflammatory condition, the two proteins contribute to resolve inflammation and tissue damage by restraining IFN-driven pro-inflammatory and apoptotic signals^7,8^. Moreover, cancer cells may highjack these negative effectors to shield themselves from IFN- or drug-induced pro-apoptotic effects^30,31^.

The inhibitory action of IFN on cell proliferation has long been recognized as being highly cell context-dependent. Accordingly, in cultured cell lines multiple pathways have been shown to be targeted by IFN, most often causing G0/G1 arrest. Several ISGs are known to exhibit growth-suppressive effect (reviewed in Sangfelt *et al*.^32^). Interestingly, it was recently reported that low level IFNβ is produced in HeLa cells during the G2/M phase and acts in an autocrine fashion to ensure an antiviral state during cell division^33^. In this regard, we have observed a small but reproducible increase in baseline levels of some ISGs in HeLa S3 cells silenced for USP18, which suggests the existence of such cell-intrinsic control. Future work is needed to assess whether low level IFNβ may tune cell cycle progression by tilting the USP18/ISG15-based control of SKP2 and possibly other cell cycle regulators.

## Materials and Methods

### Cells and culture conditions

HEK 293T, HeLa S3 and primary dermal fibroblasts from a *USP18*-deficient patient were cultured in DMEM (Gibco) supplemented with 10% fetal calf serum (PAA Laboratories, Austria), penicillin/streptomycin and 2 mM L-glutamine. Luciferase- and USP18-transduced fibroblasts from *USP18-*deficient patient were previously described^7^.

### Antibodies and reagents

We used antibodies against USP18 and AKT (Cell Signaling Technology); STAT2 (EMD Millipore); actin, V5 and Flag (Sigma-Aldrich); SKP2 and p27 (Santa Cruz Biotechnology); ISG15 (gift from E.C. Borden, Cleveland Clinic, Cleveland, OH), IFIT1 (gift from G. Sen, Cleveland Clinic, Cleveland, OH). The above antibodies are well characterized and are used by many authors in the field, including by us^7,8^. Recombinant IFN-α2b was a gift from D. Gewert (Wellcome Foundation, Beckenham, Kent, UK; now at BioLauncher Ltd, Cambridge, UK) or was purchased from Schering; IFN-β was obtained from Biogen Idec, Cambridge, MA, USA. IFNs were purified to specific activities of >10^8^ units per mg of protein and used as previously reported^7,8^. Horseradish peroxidase (HRP)-conjugated anti-rabbit IgG and anti-mouse IgG were purchased from Jackson. ALLN was from Sigma-Aldrich.

### Cell treatment and protein analyses

For IFN treatment, HeLa cells and primary dermal fibroblasts in 60-mm dishes were grown to 70–80% confluence. Subsequently, cells were treated with IFN as indicated. Whole-cell extracts were prepared by incubating cells for 45 min in lysis buffer (50 mM Tris-HCl, pH 7.4, 150 mM NaCl, 0.1% SDS, 0.5% deoxycholate, 1 mM EDTA, 1% NP40, 1x complete protease inhibitor cocktail, 0.1 mM Na3VO4 and 10 mM NaF) on ice. The suspension was centrifuged at 12,000 g for 10 min at 4 °C. The protein content was measured by standard Bradford assay protocol. Proteins were denaturated by boiling at 95 °C for 5 min with 6X sampling buffer. An aliquot of 35 μg of proteins were separated by SDS-PAGE and transferred onto nitrocellulose membrane (Amersham GE Healthcare Life Science). Then, membranes were stained with Red Ponceau and analyzed by western blot as detailed in the supplementary information section. For western blotting, membranes were blocked with 5% non-fat dried milk in PBS Tween 0.1% for 1 h and then incubated with the first antibody at 4 °C overnight. After 3 washings, membranes were incubated with HRP-conjugated secondary antibody. Signal was detected with an enhanced chemiluminescence (ECL) detection reagent (SuperSignal^®^ West Pico Chemiluminescent Substrate, Thermoscientific). Images were acquired with a Fuji ImageQuant LAS-4000 machine for time periods of 10 sec during 2 min. After this time, if the signal was low or absent, the membrane was rinsed in PBS Tween 0.1% and a 50/50 blend of SuperSignal^®^ West Pico and SuperSignal^®^ West Femto chemiluminescent substrates (Thermo Scientific) and images were acquired as above. The data were analyzed by using Multi Gauge v3.2 (Fuji Image Analyzing Application) software. The expression levels of protein were normalized by that of the corresponding β actin or GAPDH as indicated in the figures. In some cases, membranes were stripped with Restore^TM^ Western Blot Stripping buffer (Thermo Scientific) was according the manufacturer’s instructions and reprobed for detection of actin or GAPDH.

For co-immunoprecipitation assay, cells were lysed in 50 mM Tris pH 7.5, 200 mM NaCl, 1 mM EDTA, 0.5% NP40, 10% glycerol, 1x complete protease inhibitor cocktail, 0.1 mM Na3VO4 and 10 mM NaF). The supernatant was separated by centrifugation at 12,000 g at 4 °C for 10 min and pre-cleared with protein G/protein A-agarose (Calbiochem). The supernatant was collected by centrifugation at 2,000 g at 4 °C for 3 min and incubated with antibody as indicated for 3 h at 4 °C. Complexes were precipitated with protein G/protein A-agarose, washed and resuspended in SDS sample buffer. Immunoprecipitates were subjected to SDS-PAGE and western blotting, as described above. For the analysis of SKP2 ISGylation, cells were incubated in lysis buffer and SKP2 was immunoprecipitated overnight at 4 °C. Complexes were precipitated with protein G/protein A-agarose, washed and resuspended in SDS sample buffer. Immunoprecipitates were subjected to SDS-PAGE and western blotting, as described above.

### siRNA transfection

In all experiments, the *ISG15*-targeting siRNA, *ISG15#12* (GCAACGAAUUCCAGGUGUC) (Sigma) and a control non-targeting pool siRNA from Dharmacon were used as reported^8^. Three *USP18*-targeting siRNAs (Sigma) were individually used: *USP18#7* (CUGCAUAUCUUCUGGUUUA); *USP18#9* (GGACUACCCUCAUGGCCUG) and #12 (GGAAUUCACAGACGAGAAA). HEK 293T and HeLa S3 cells were transfected with siRNAs using Lipofectamine RNAiMAX Transfection Reagent and Reverse Transfection protocol (Invitrogen) according to the manufacturer’s instructions. Briefly, 2.2 μl siRNA of a 50 μM stock and 3.8 μl of Lipofectamine RNAiMAX were separately diluted in 500 μl Opti-MEM reduced serum medium (Invitrogen) and incubated for 5 min at room temperature. Then, diluted Lipofectamine RNAiMAX was added to RNAi molecules and mixed together. After 20 min-incubation at room temperature the transfection mix was placed in 60 mm petri dish and 0.45 × 10^6^ cells in 2 ml were seeded on top.

### Plasmids and transfection

HEK 293T cells were transiently transfected with FuGENE6 (Roche Applied Science) complexed with different constructs according to manufacturer instructions. The following constructs were used for expression, co-immunoprecipitation and immunoprecipitation assays: pcDNA3-flag-SKP2 (from E. Bianchi, Institut Pasteur, Paris, France), pcDNA3-3xflag-huISG15 (from J. Huibregtse, University of Texas at Austin, TX, USA) from which we derived the non conjugatable ΔGG mutant, pcDNA4b-huUSP18^34^, pcDNA4b-huUSP18-V5, pcDNA4-UbE1L, pcDNA4-UBCH8, pCMV-Sport6-EFP, Myc-HERC5. The pcDNA4b-huUSP18 I60N-V5 and C64S mutants, pcDNA4b-huUSP18-V5 long and short were all derived from the construct above. SKP2 mutants were generated by mutating lysine into arginine residues as indicated in Fig. S1A, using Quick-change site directed mutagenesis kit (Agilent Technologies Inc.). Plasmids were re-sequenced.

### Cell cycle analysis

For synchronization, HeLa S3 cells were arrested in G1/S boundary by double thymidine block and release as previously reported^35^. Briefly, cells were first incubated with 2.5 mM Thymidine (Sigma) for 16 h and then released for 8 h. The thymidine block was repeated and the second release was performed for the indicated period of time. Cells were collected, washed with PBS, fixed in cold 70% ethanol, and resuspended in PBS containing 50 μg/mL propidium iodide (Sigma) and 200 μg/mL RNase A (Sigma). Analyses were performed with a cytoFLEX S flow cytometer (Beckman Coulter) and FlowJo v10.0 software.

### Apoptosis assay

HeLa S3 cells were synchronized as described above. Cells were collected, and prepared for flow cytometric analysis of DNA content and PARP cleavage according the permeabilization protocol provided by Cell Signaling Technology with few modifications. Briefly, cells were fixed in paraformaldehyde 4% for 15 min and kept in ice-cold 90% ethanol for at least 30 min and stored at −20 °C. Ethanol was removed by centrifugation and the cell pellet was washed twice with PBS. Immunostaining with anti-cleaved PARP antibody was performed in 0.5% (w/v) solution of bovine serum albumin in PBS for 2 h at room temperature. Then, cells were washed and incubated with APC-conjugated secondary antibody for 1 h at room temperature in the dark. Cells were washed and the cell pellet was incubated with propidium iodide as described above for DNA content determination. The proportion of cells expressing cleaved PARP and DNA content was analyzed using a cytoFLEX S flow cytometer (Beckman Coulter) and FlowJo v10.0 software.

## Supplementary information

Supplementary information
