# Supplementary material for: USP18 and ISG15 coordinately impact on SKP2 and cell cycle progression
Source: Sci Rep. 2019 Mar 11;9:4066. doi: 10.1038/s41598-019-39343-7 (PMC6411882; doi:10.1038/s41598-019-39343-7)

SUPPLEMENTARY INFORMATION

USP18 and ISG15 coordinately impact on SKP2 and cell cycle progression

Françoise Vuillier[1], Zhi Li[1], Pierre-Henri Commere[2], Lasse Toftdal Dynesen[1], and Sandra Pellegrini[1]

[1]Institut Pasteur, Unit of Cytokine Signaling, Inserm U1221, 75724 Paris, France; [2]Institut Pasteur, Flow Cytometry Platform, 75724 Paris, France.

Legend to Supplementary Fig. S1

ISG15 abrogates the USP18-SKP2 complex regardless of its conjugation capacity.

293T cells were transfected with Flag-SKP2 in association with USP18-V5 and/or 3xFlag-ISG15 WT or ΔGG, as indicated. Anti-V5 immunoprecipitates (top) and total lysates (bottom) were subject to immunoblot with antibodies to SKP2, V5 and Flag. By co-immunoprecipitation we confirmed the binding of SKP2 (55 kDa) to USP18 (Fig. 1A, lane 1). When ISG15 was co-expressed, no such complex was detected and instead ISG15 was found to bind USP18 (lane 3).

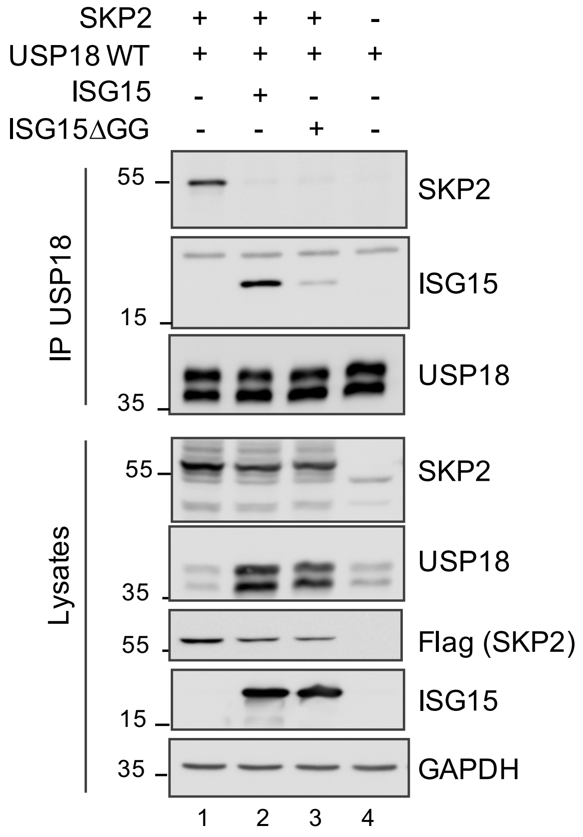

Supplementary Fig. S1

Legend to Supplementary Fig. S2

Free ISG15 promotes SKP2 ubiquitination.

293T cells were transiently transfected with HA-ubiquitin alone or in the presence of Flag-ISG15 WT or ΔGG, as indicated. To ease detection of ubiquitinated species, N-ethylmaleimide (NEM, 10µM) was added 6 hr before cell lysis (Liu et al, *J. Biol. Chem,* 2003. 278:1594). Endogenous SKP2 was immunoprecipitated and an irrelevant antibody was used as control (IP ctrl). Immunoprecipitates (left) and lysates (right) were analyzed by immunoblot with the indicated antibodies.

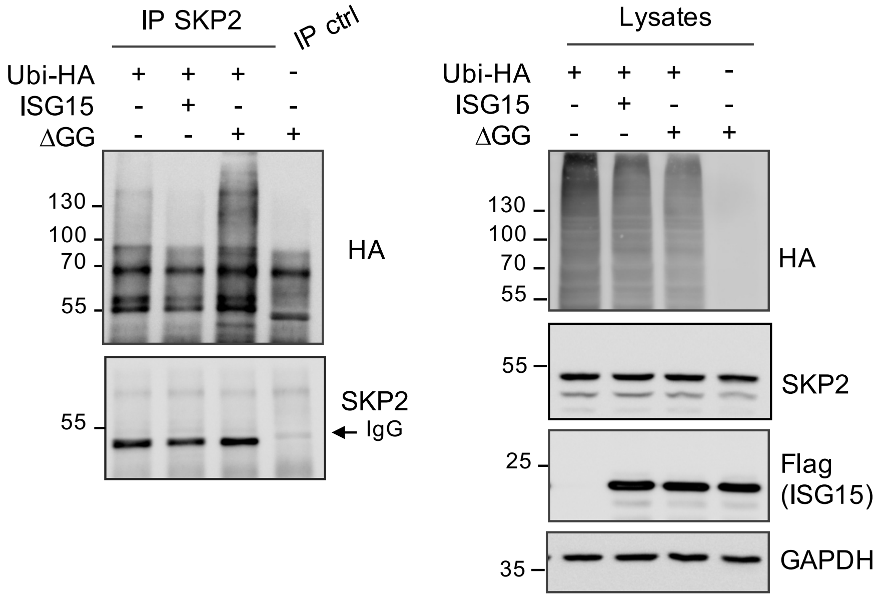

Supplementary Fig. S2

Legend to Supplementary Fig. S3

ISGylation of Lys to Arg SKP2 mutants.

(**A**) Schematic representation of all Lys residues in SKP2 that have been substituted with Arg. The underlined Lys have been mutated together. The asterisks indicate lysine residues for which the corresponding mutant was not tested for their ability to be ISGylated. LRR, Leucine Rich Repeats ; NLS, Nuclear Localization Sequence.

(**B**) Expression levels of SKP2 mutants in HEK 293T cells. Cells were transfected with SKP2 wild type (WT) or mutants, as indicated. Two days later, levels of SKP2 protein were analyzed in cell lysates by immunoblotting with anti-flag antibody.

(**C**) ISGylation of SKP2 mutants. HEK 293T cells were transfected as in (B). Two days later, lysates were subjected to immunoprecipitation (IP) with anti-SKP2 antibodies. Immunoprecipitates were immunoblotted by anti-ISG15 antibodies as indicated. The membrane was cut above the 55kDa band corresponding to the Ig heavy chain, as indicated by a dotted line. Whole cell lysates were subjected to immunoblot with anti-SKP2 or anti-flag antibodies to analyze SKP2 expression level.

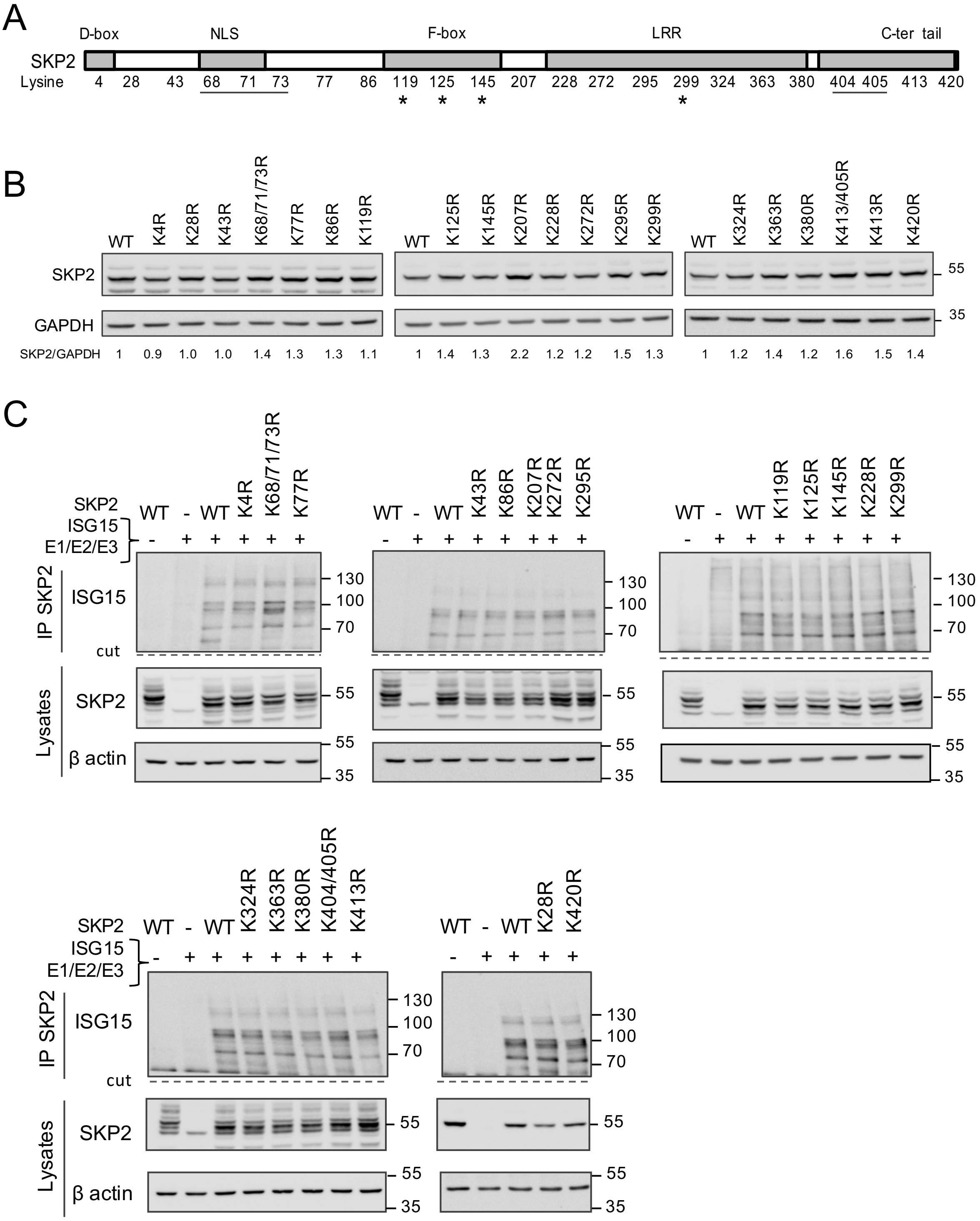

Supplementary Fig. S3

Legend to Supplementary Fig. S4

Effect of two additional USP18 siRNA.

(A) Impact of silencing USP18 with two additional siRNAs (#9 and #12) on SKP2 level. HeLa S3 cells were retro-transfected with control siRNA (siCTRL) or the indicated siRNA targeting USP18. Cells were left untreated or treated with IFNβ for 24 hr. Lysates were analyzed by western blot with anti-SKP2, anti-USP18 or anti-ISG15 antibodies. Boxed lanes: 20x less cell lysate was loaded. The intensity of SKP2 bands (upper blot) were normalized to that of GAPDH (bottom blot). Results are reported as the ratio of SKP2 to GAPDH. The ratio obtained for unstimulated cells was set to 1. Long exposure: time extended 3x, after hiding the right portion of the membrane.

(B) Impact of silencing USP18 with siRNAs #9 and #12 on cell cycle progression. HeLa S3 cells were retro-transfected with control siRNA (siCTRL) or the two indicated siRNA targeting USP18. Cells were left in asynchronous state (AS) or synchronized in G1/S and released into fresh medium as described in Figure 5. Progression through the cell cycle was monitored for the first 4 hr after the release. Cells were stained with propidium iodide for DNA content and the cell cycle profile was determined by flow cytometry. Debris and sub-2N phase material were excluded. G0G1 phase (2N) is indicated. Values shown in each histogram represent the percentage of cells in G0/G1 phase.

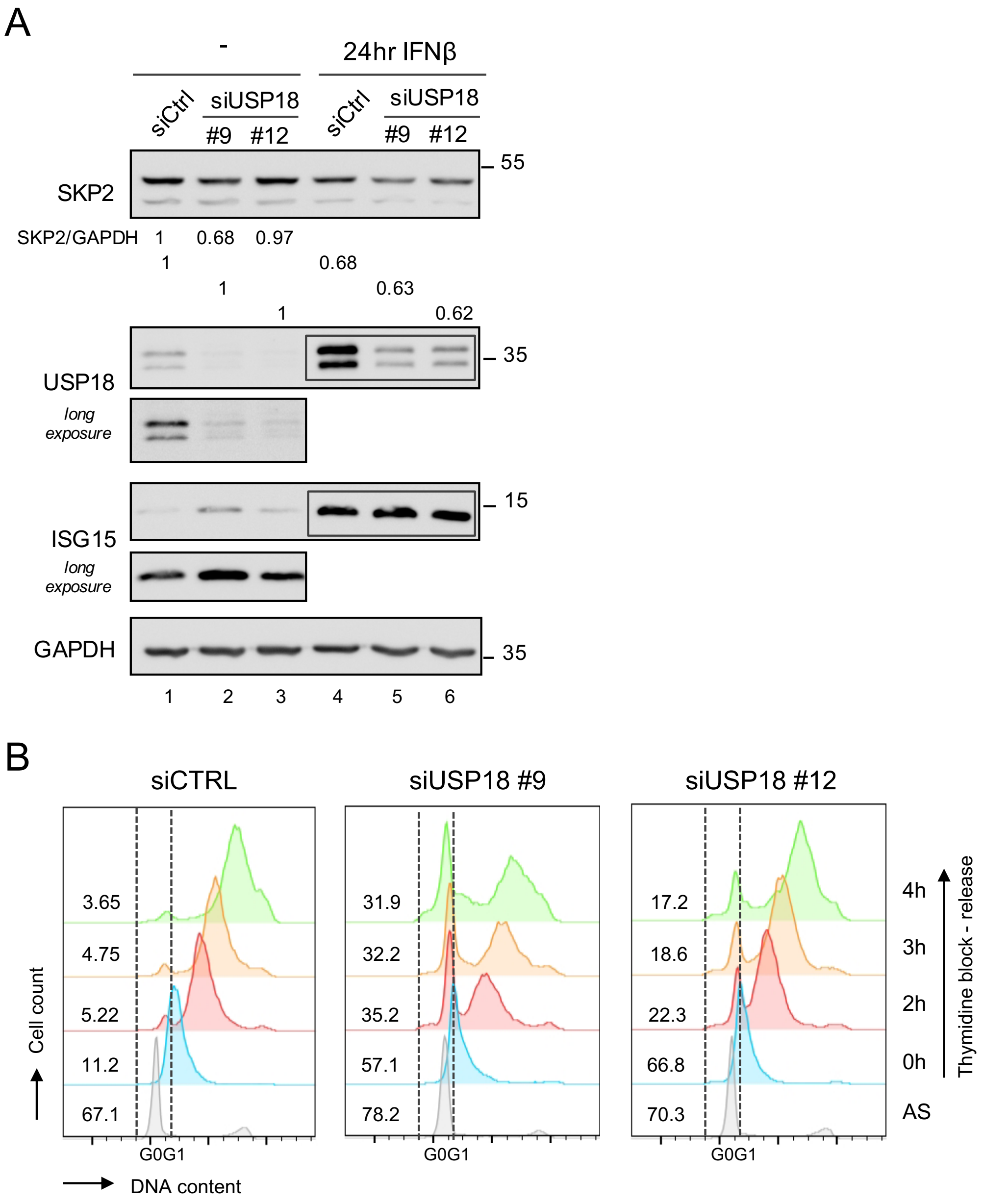

Supplementary Fig. S4

Supplementary Fig. S5

Full-length western blot of Figure 1A

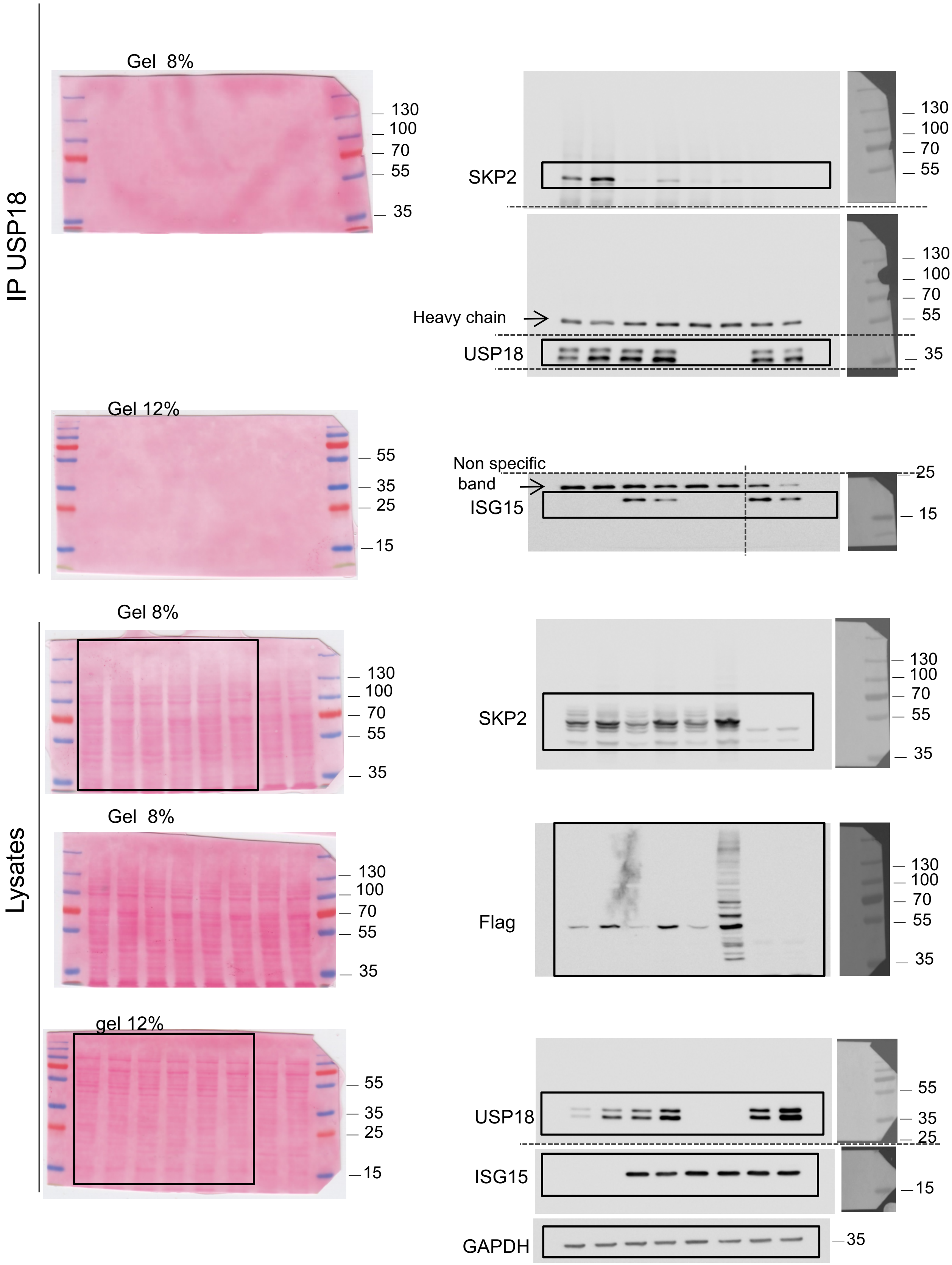

Supplementary Fig. S6

Full-length western blot of Figure 1B

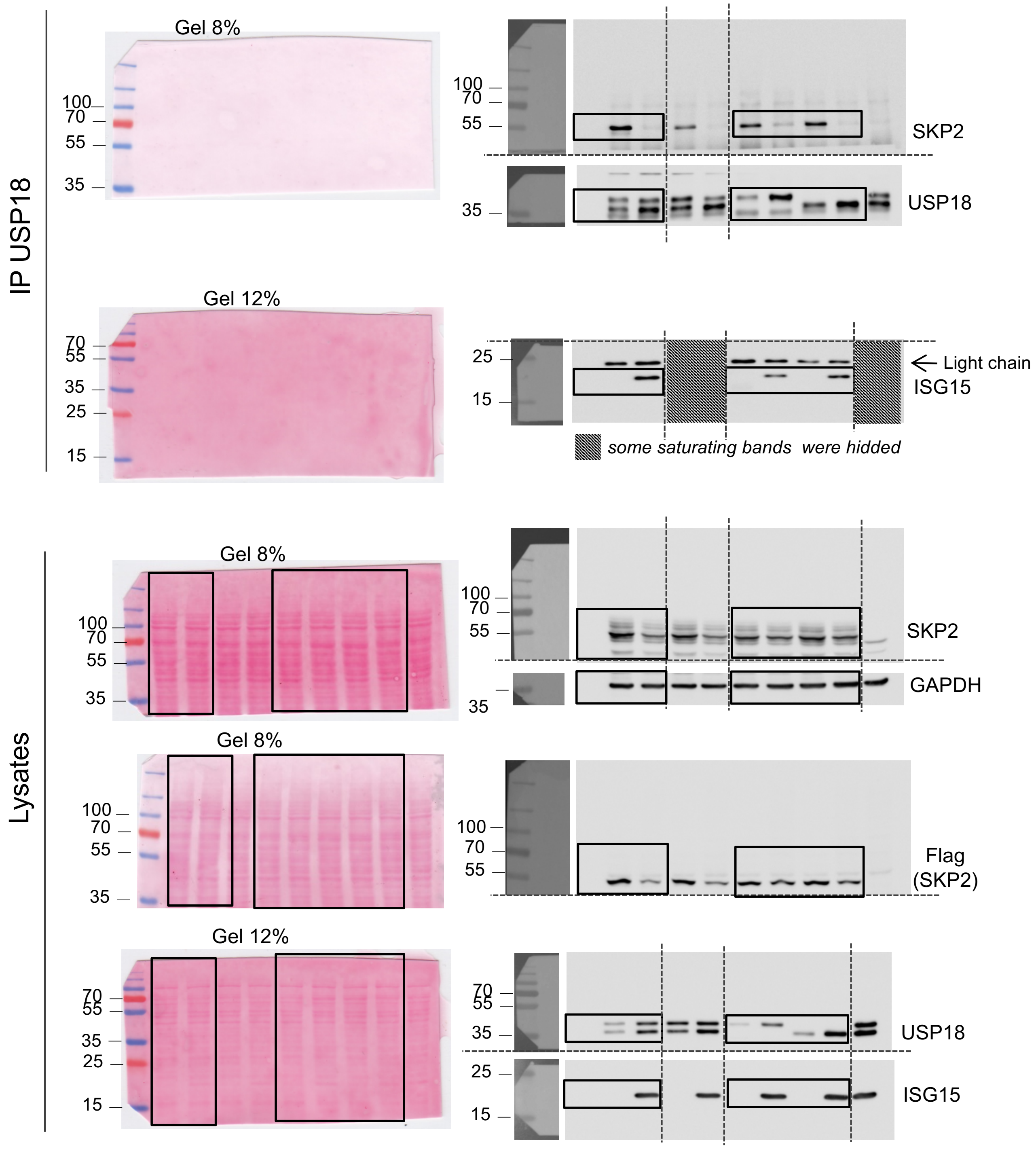

Supplementary Fig. S7

Full-length western blot of Figure 1C

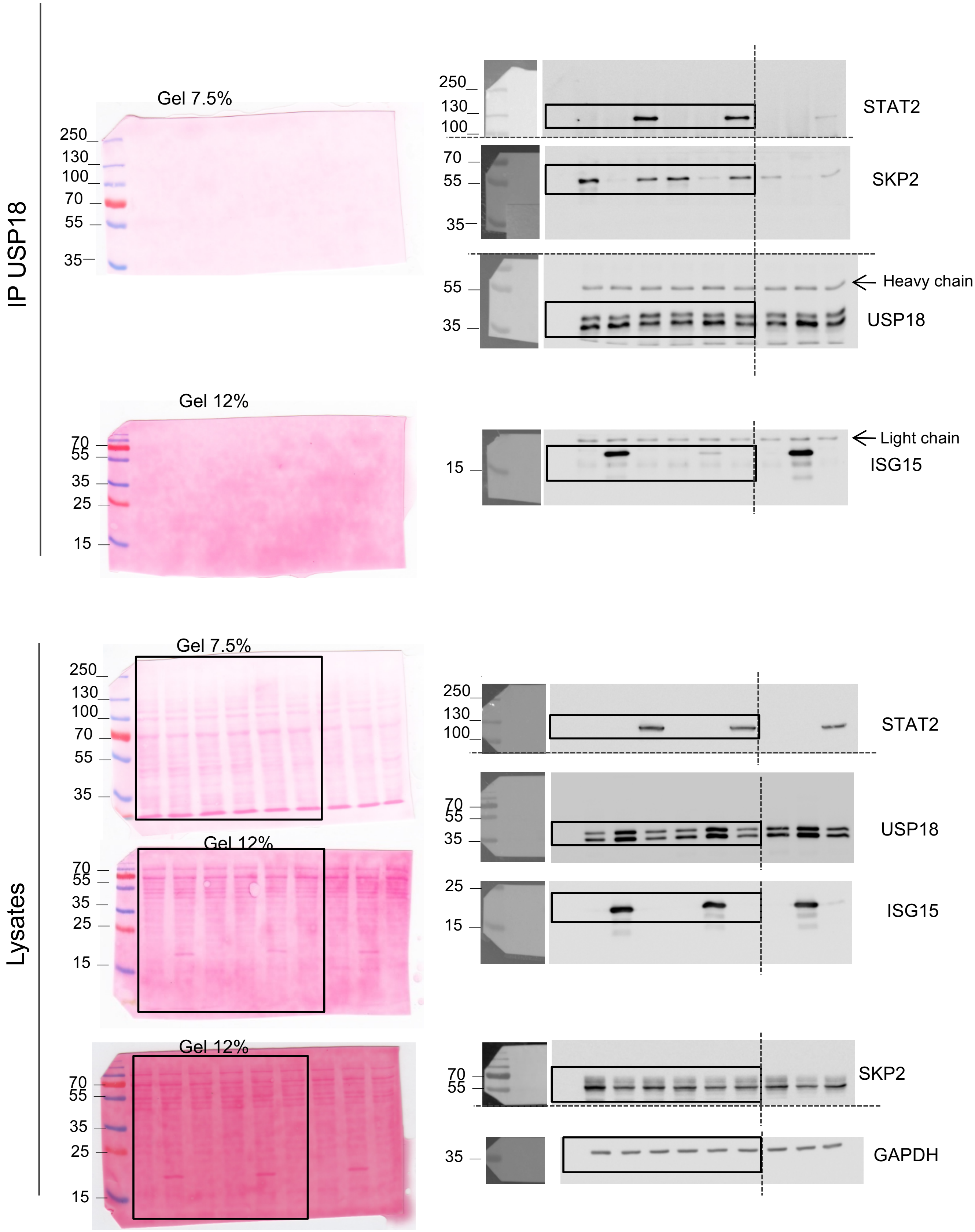

Supplementary Fig. S8

Full-length western blot of Figure 1D

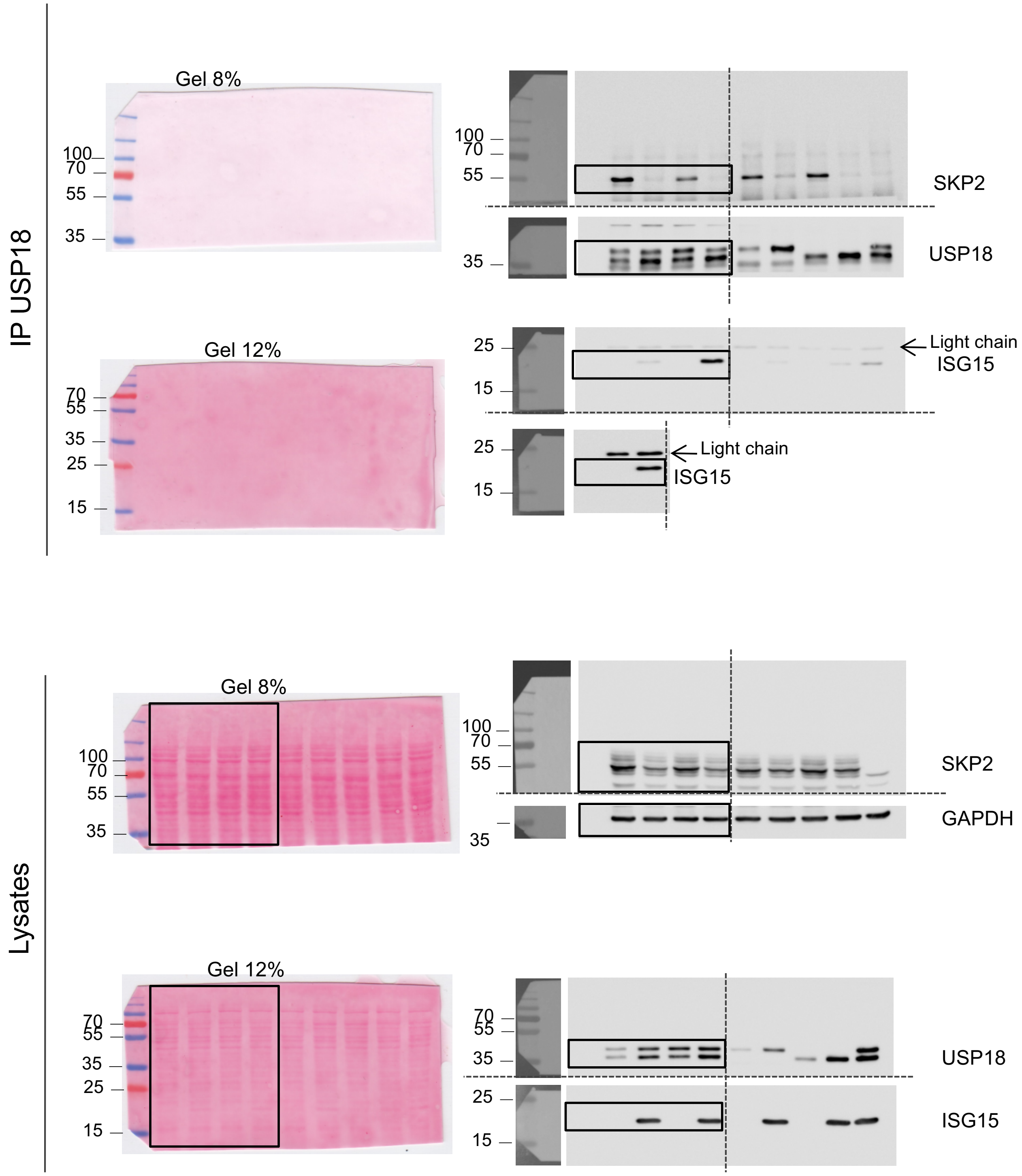

Supplementary Fig. S9

Full-length western blot of Figure 2A and 2B

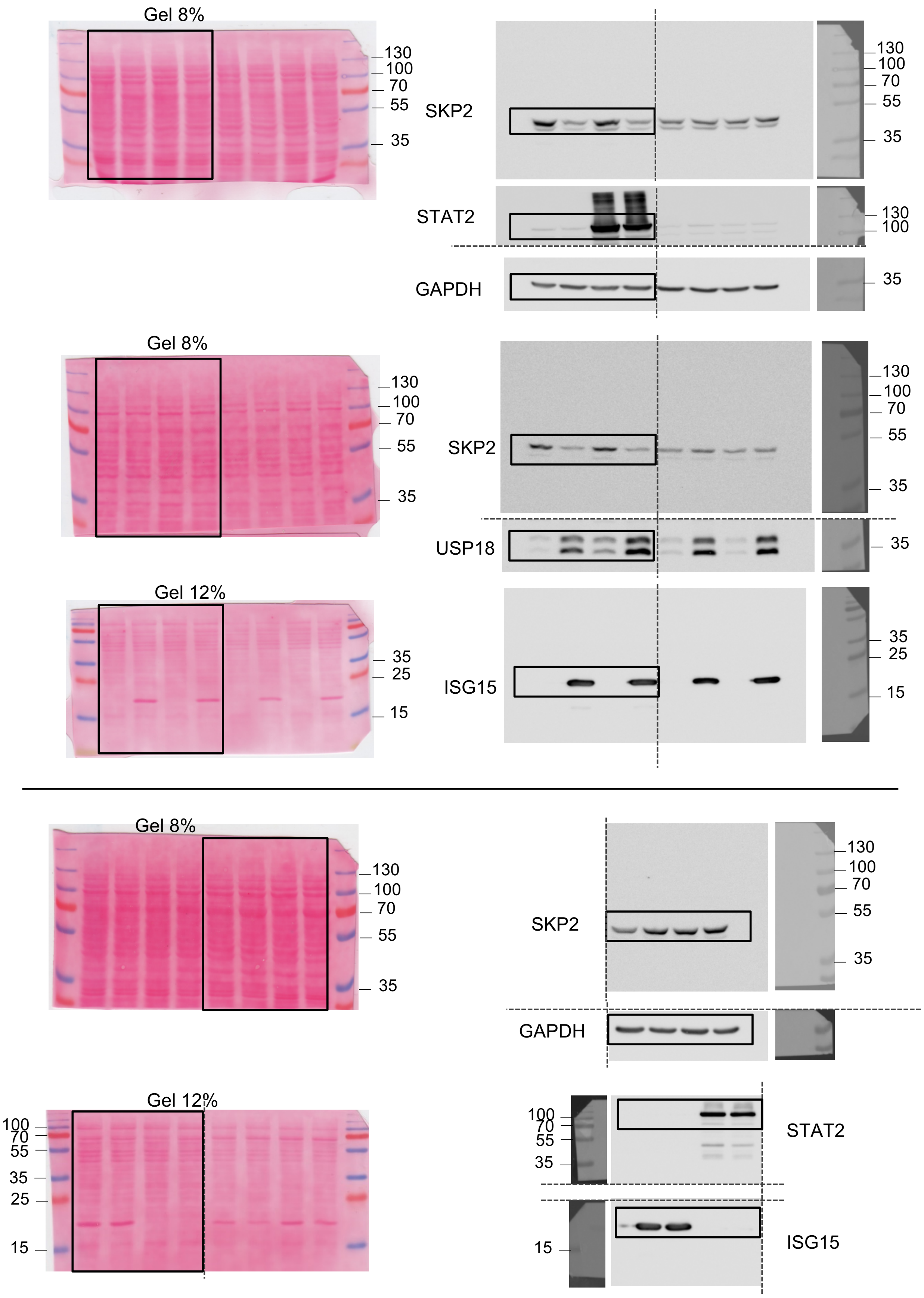

Supplementary Fig. S10

Full-length western blot of Figure 3A

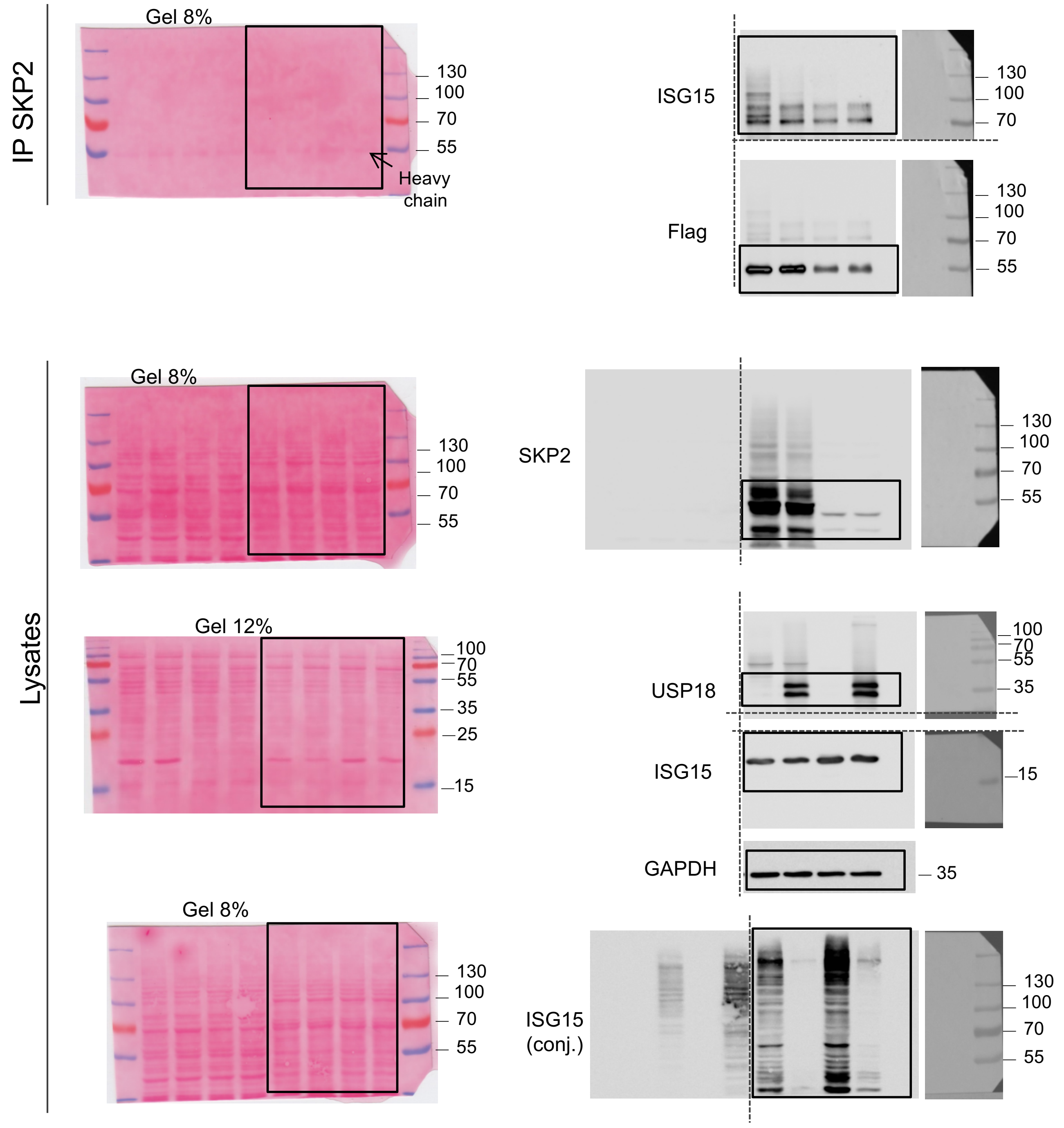

Supplementary Fig. S11

Full-length western blot of Figure 3B

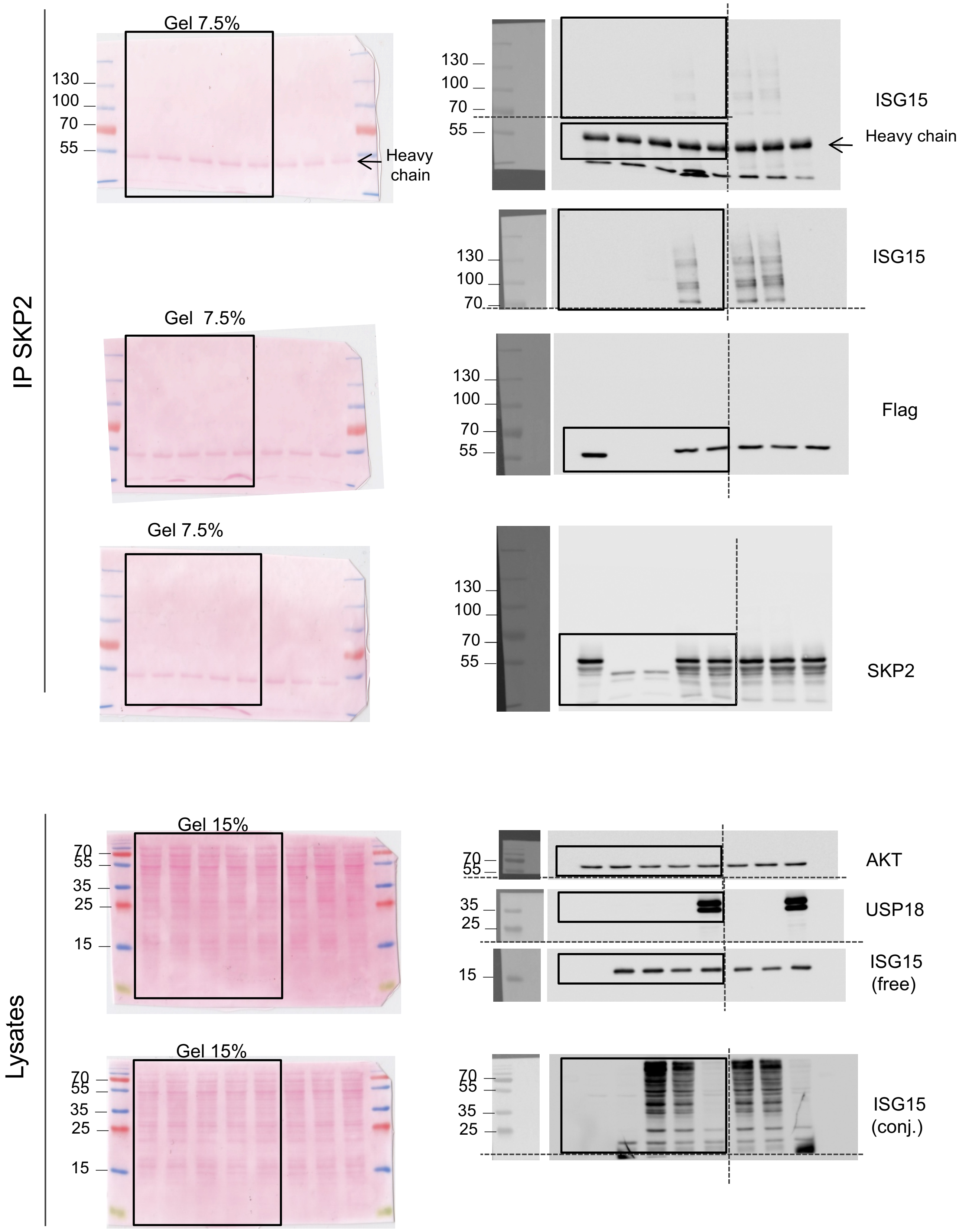

Supplementary Fig. S12

Full-length western blot of Figure 3C

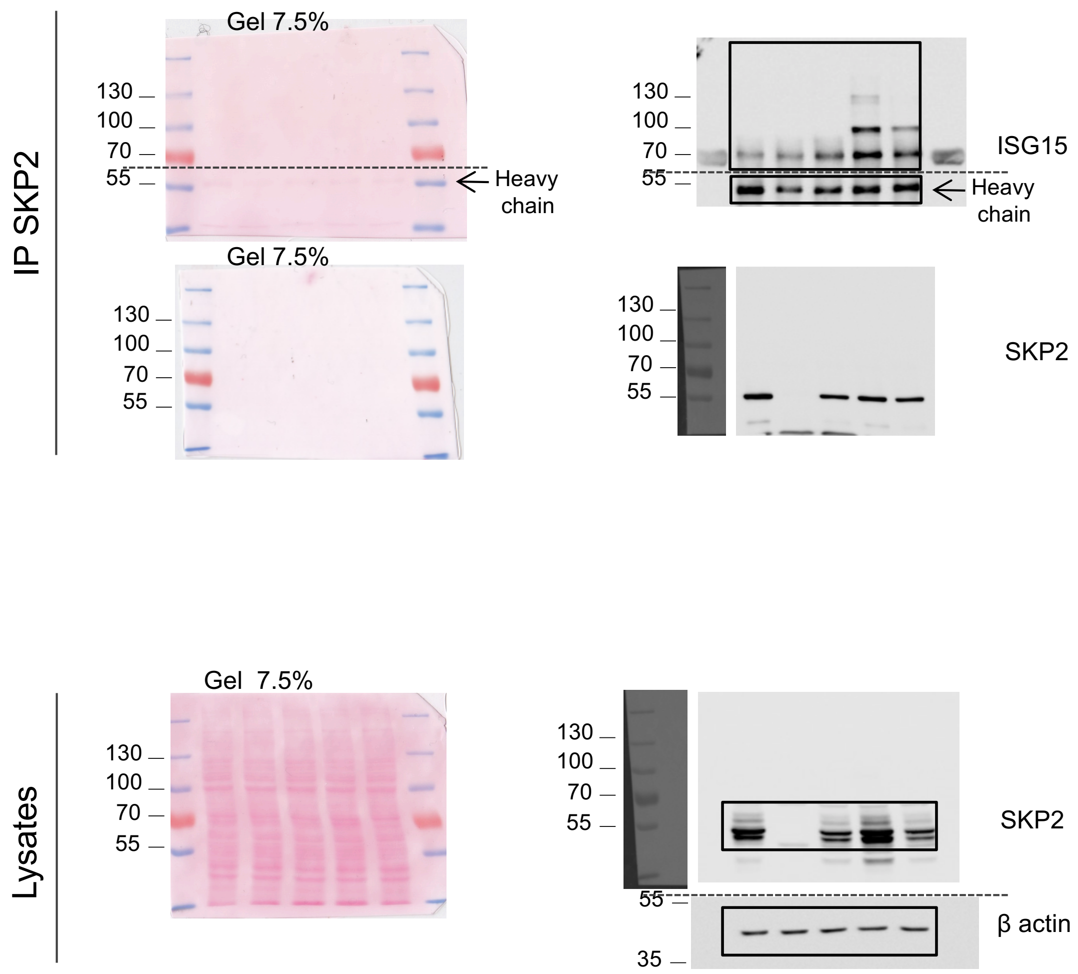

Supplementary Fig. S13
Full-length western blot of Figure 3D (IP panel)

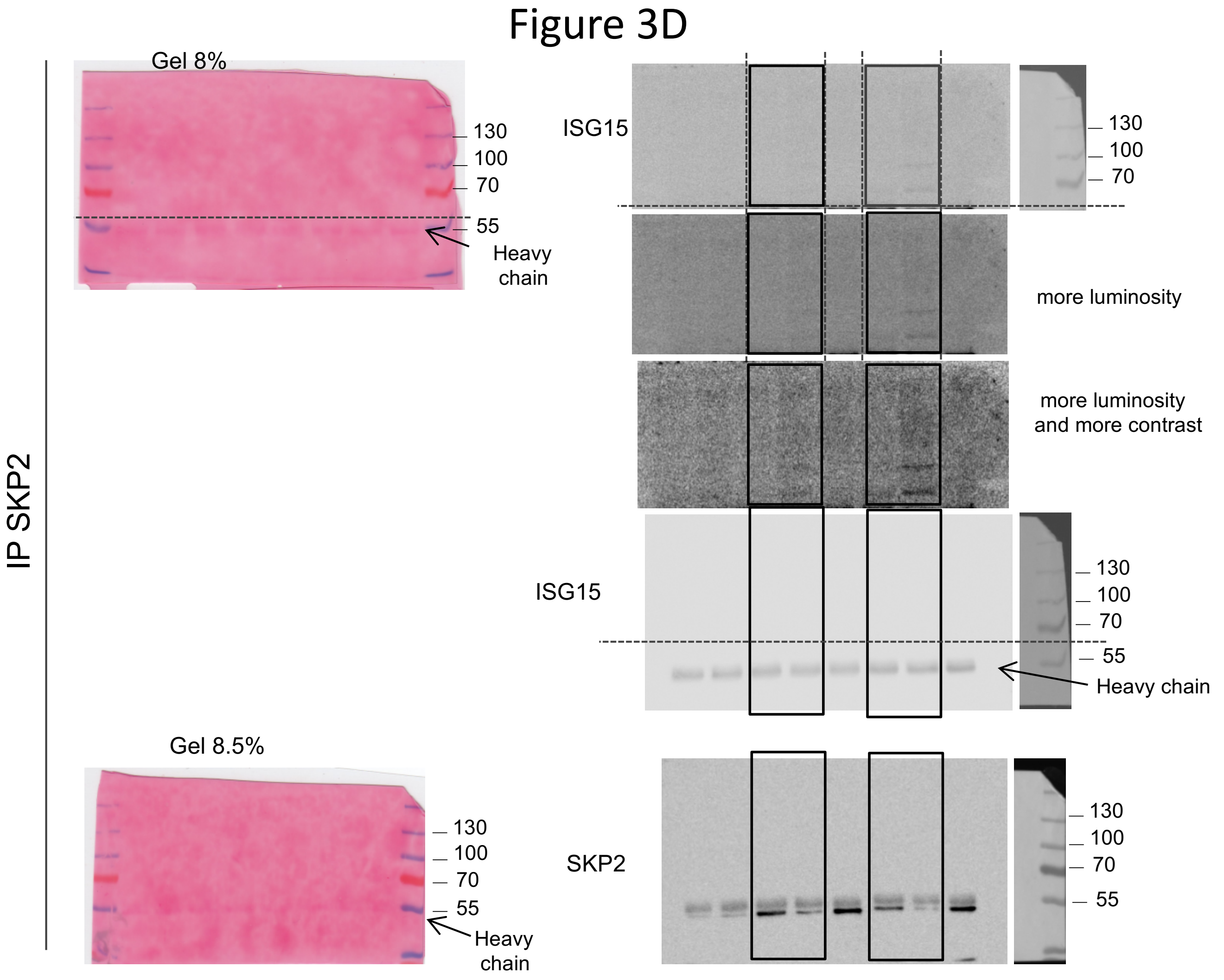

Supplementary Fig. S14

Full-length western blot of Figure 3D (Lysates panel)

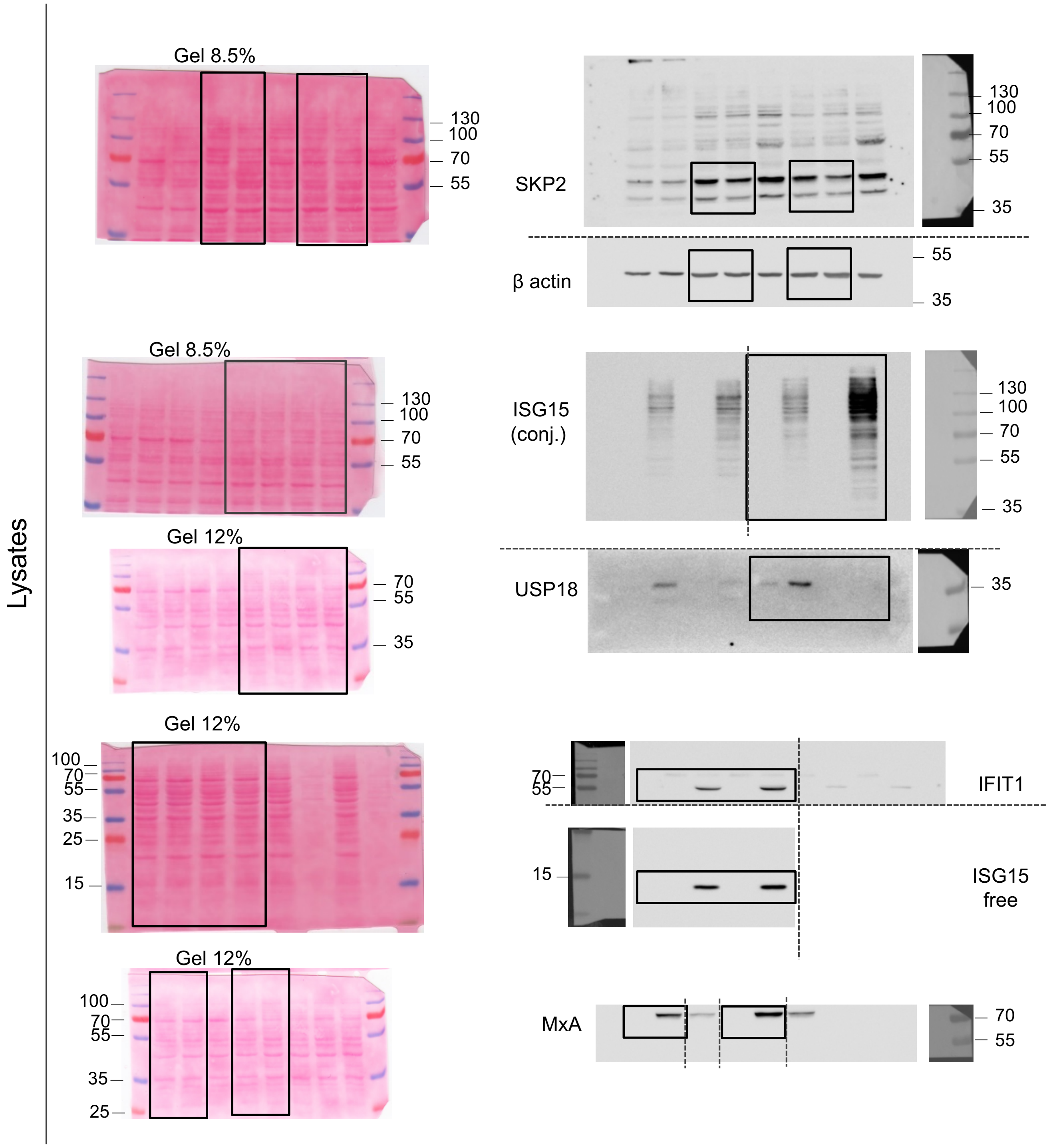

Supplementary Fig. S15

Full-length western blot of Figure 4A and 4B

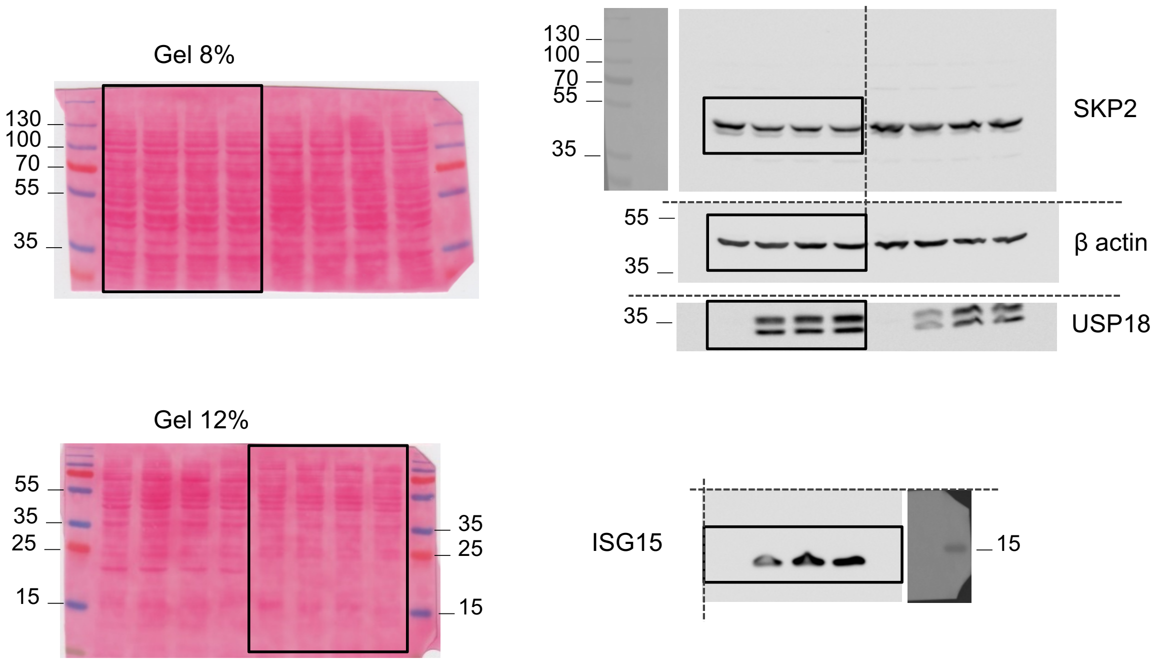

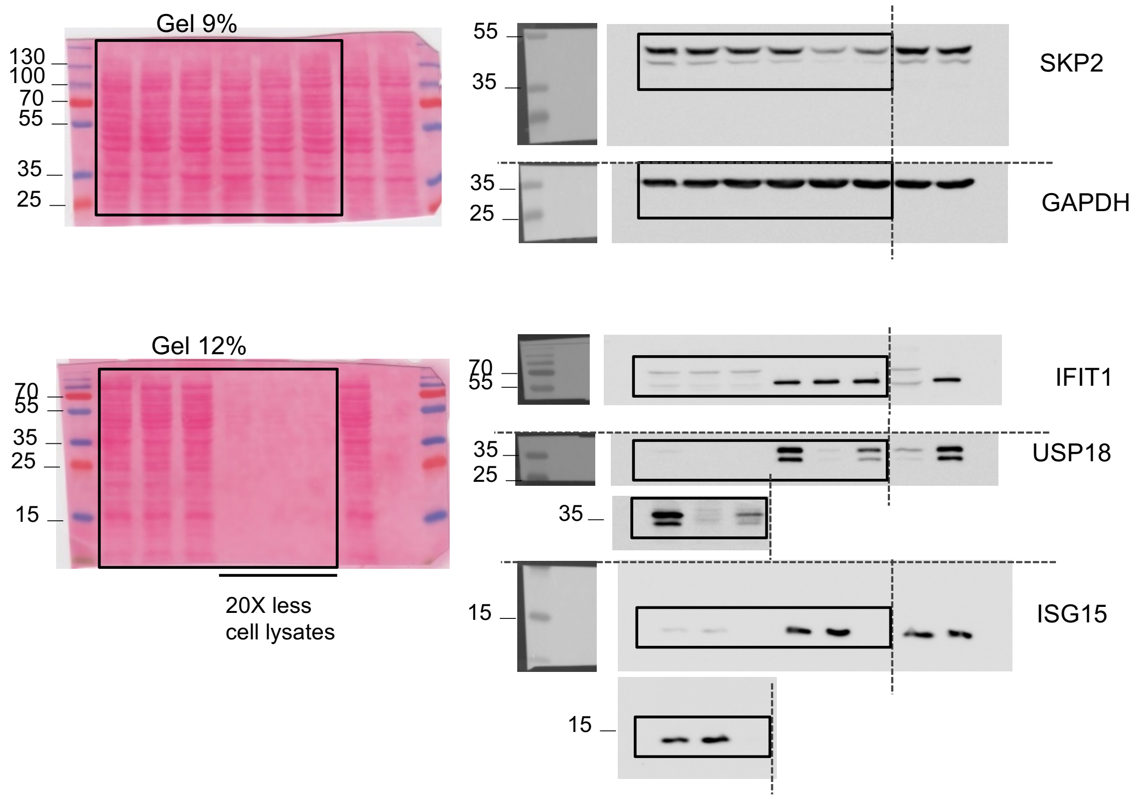

Supplementary Fig. S16

Full-length western blot of Figure 5C and 6A

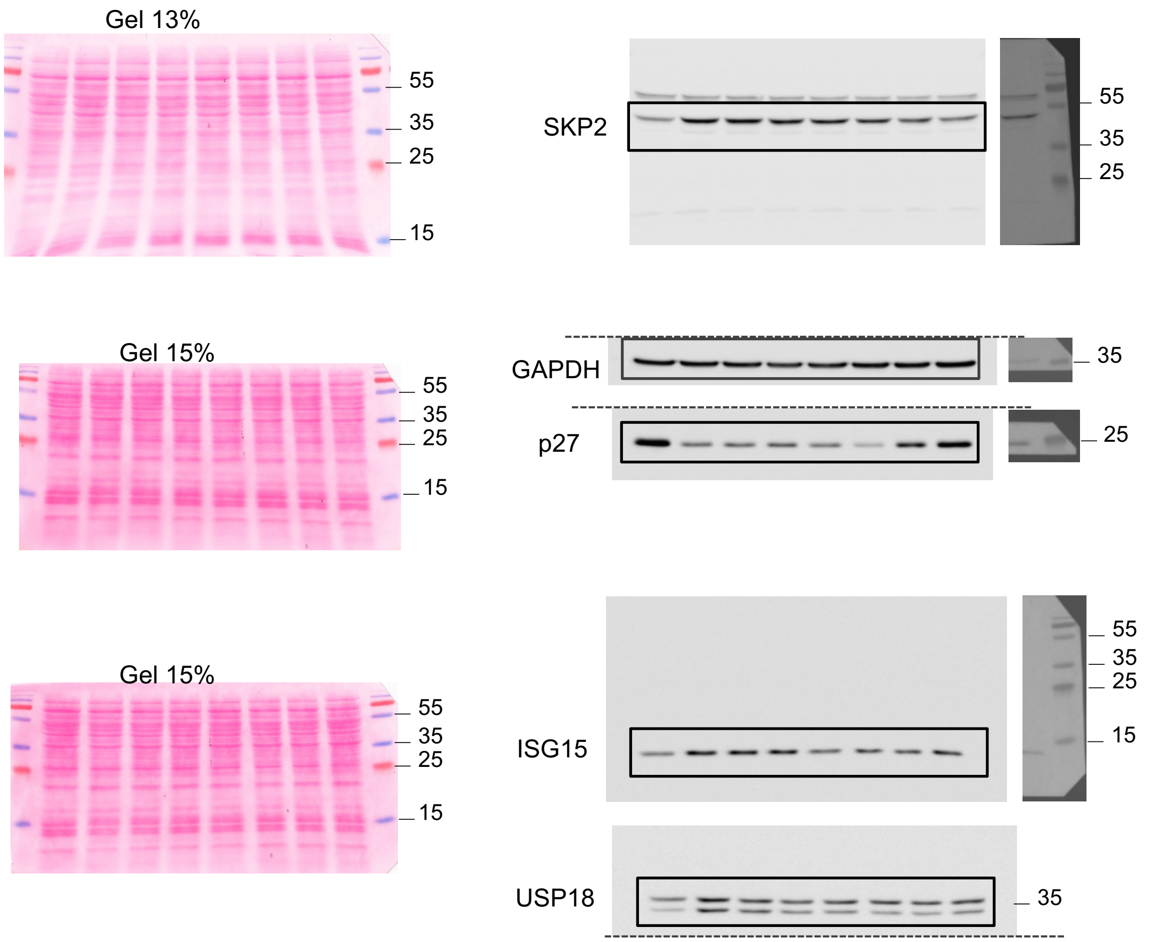

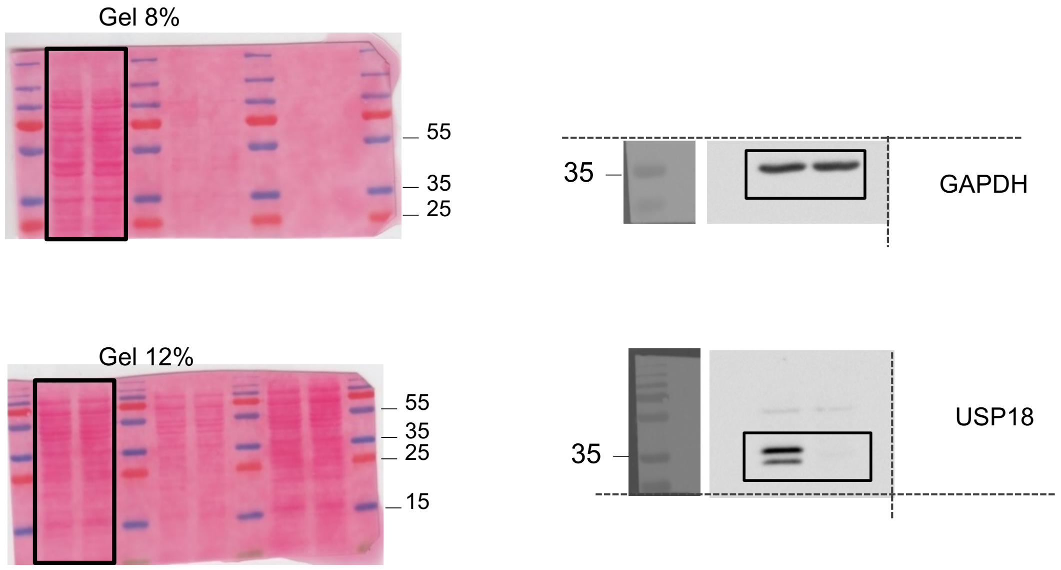

Supplementary Fig. S17

Full-length western blot of Figure S1

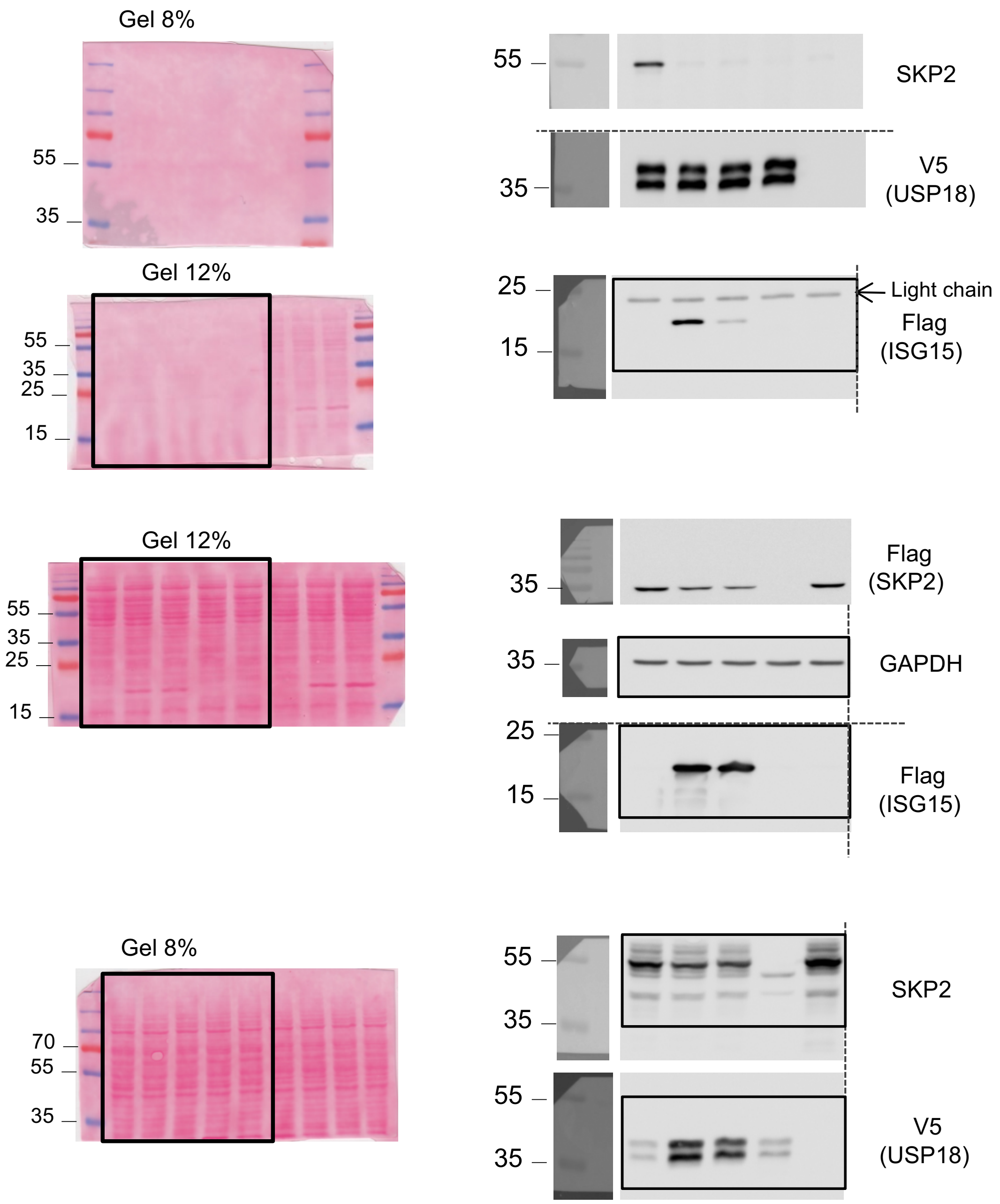

Supplementary Fig. S18

Full-length western blot of Figure S2

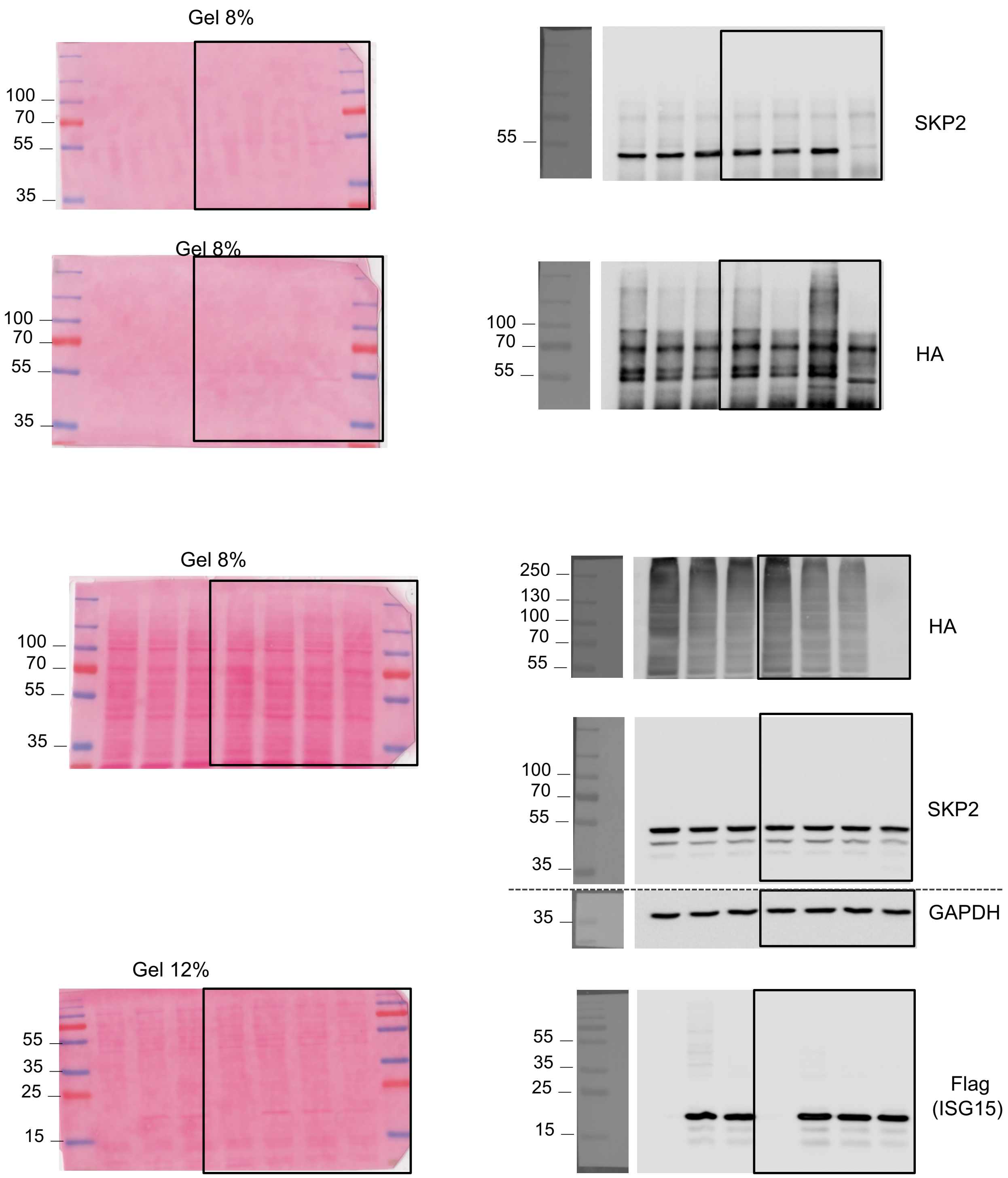

Supplementary Fig. S19

Full-length western blot of Supplementary Fig. S3B

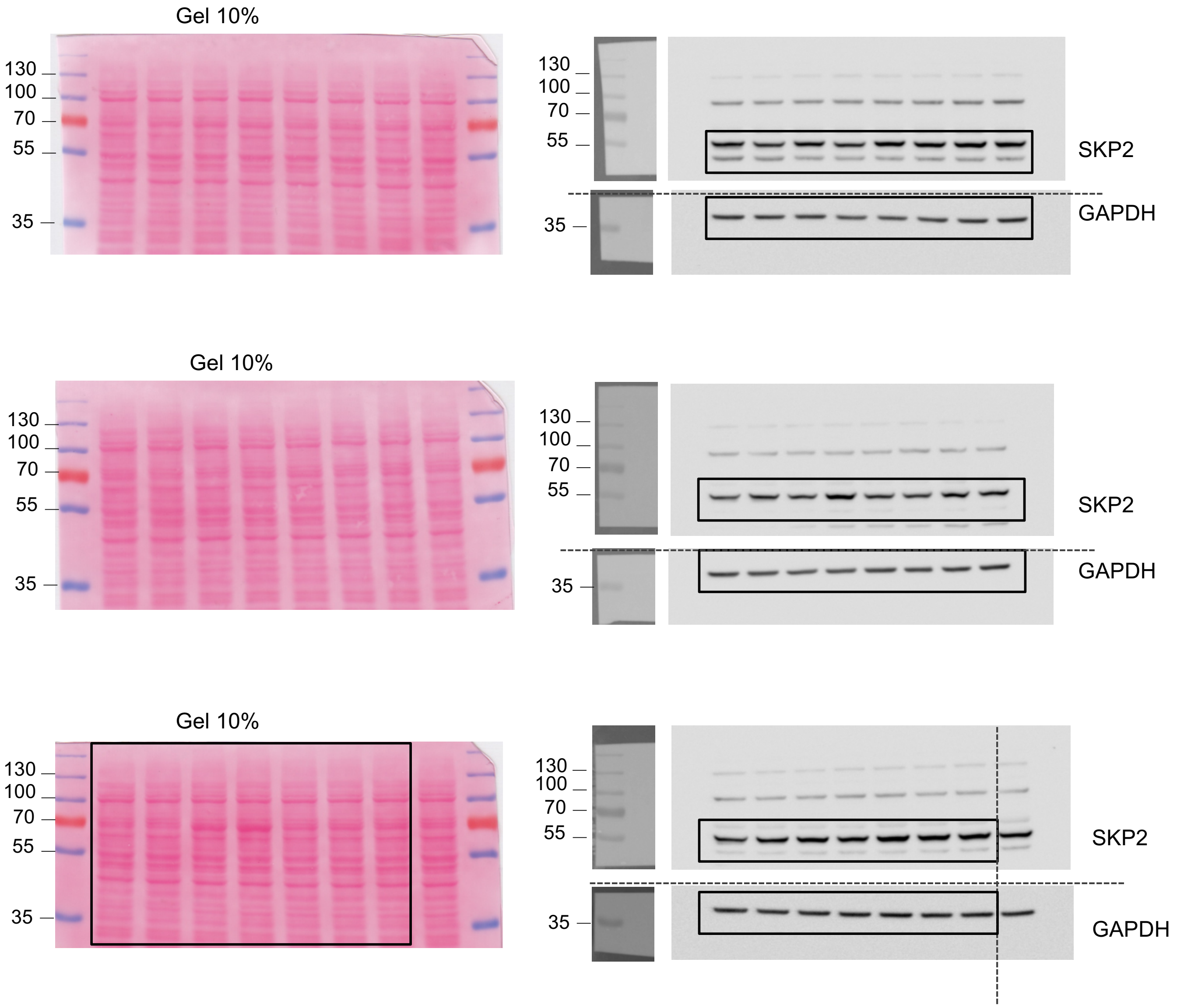

Supplementary Fig. S20
Full-length western blot of Supplementary Fig. S3C (panels 1 and 2)

Panel #1

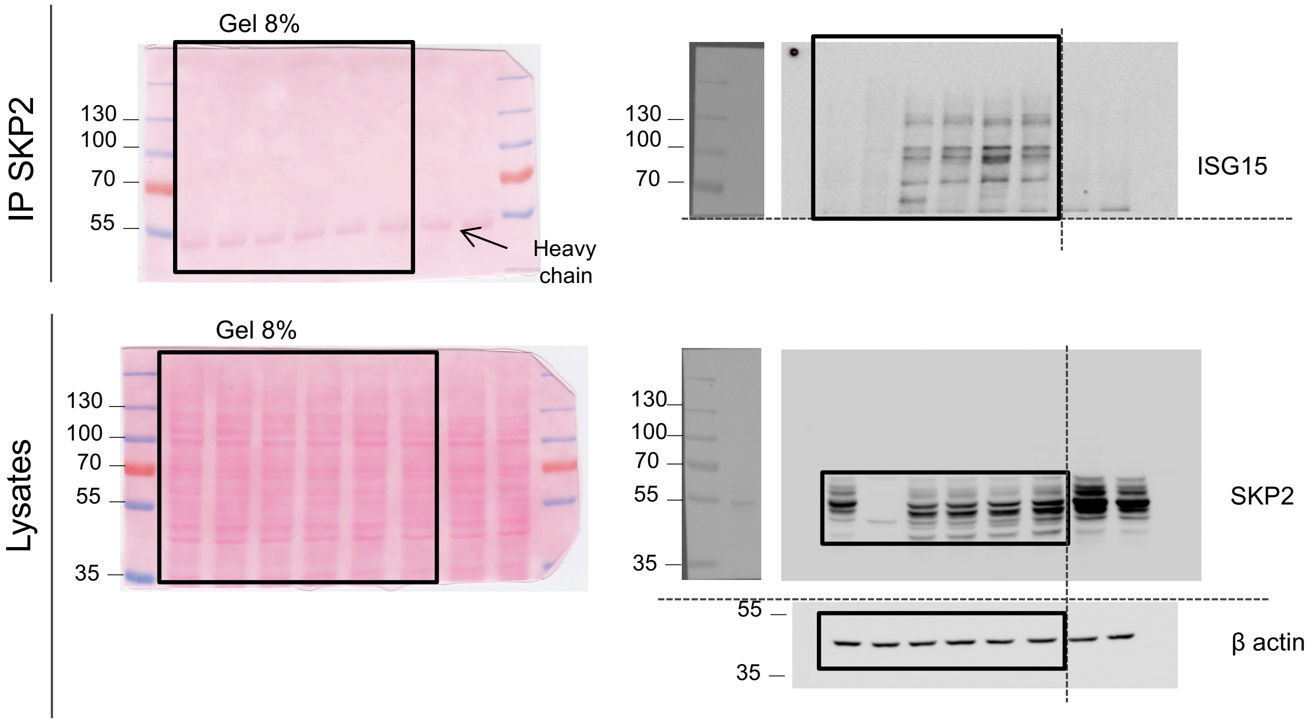

Panel #2

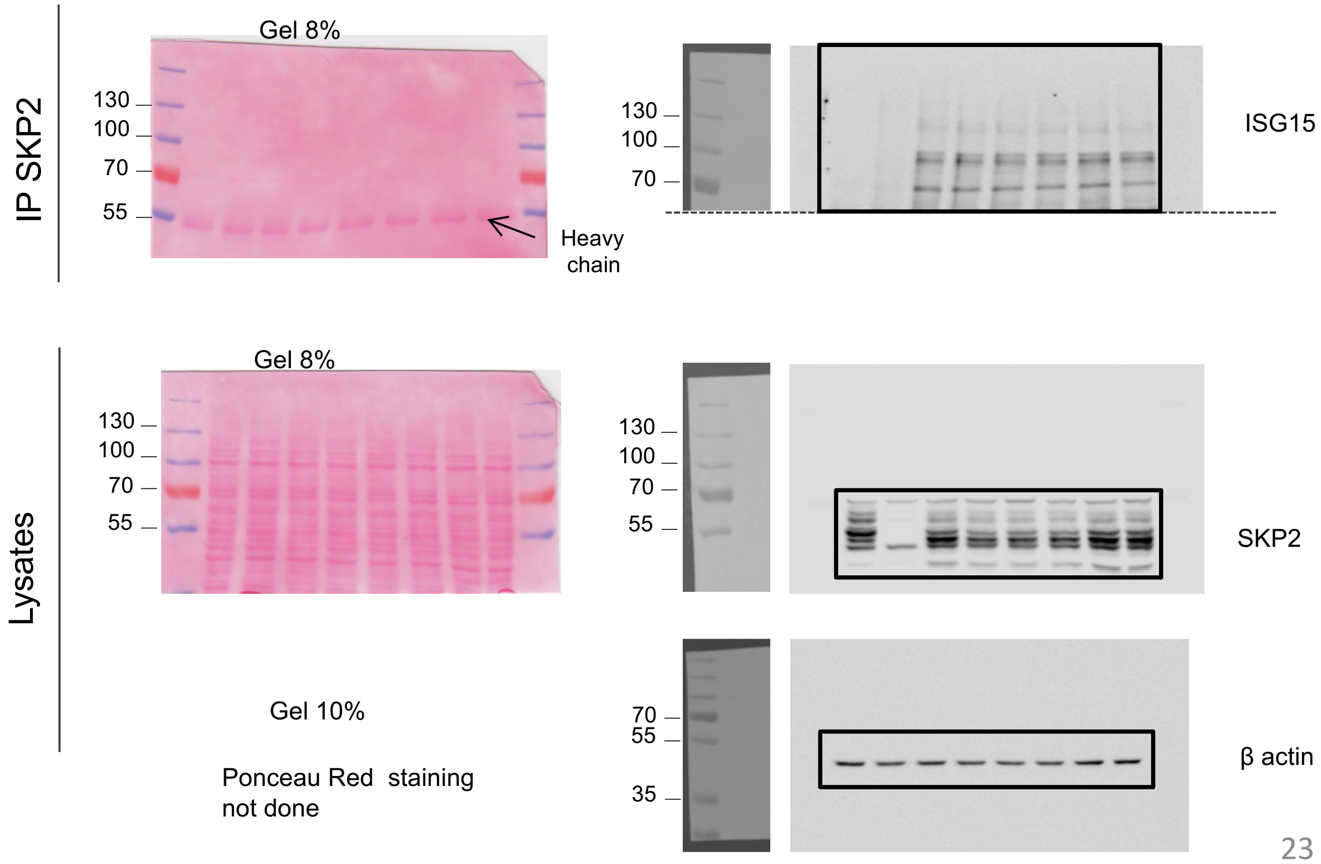

Supplementary Fig. S21

Full-length western blot of Supplementary Fig. S3C (panels 3 and 4)

Panel #3

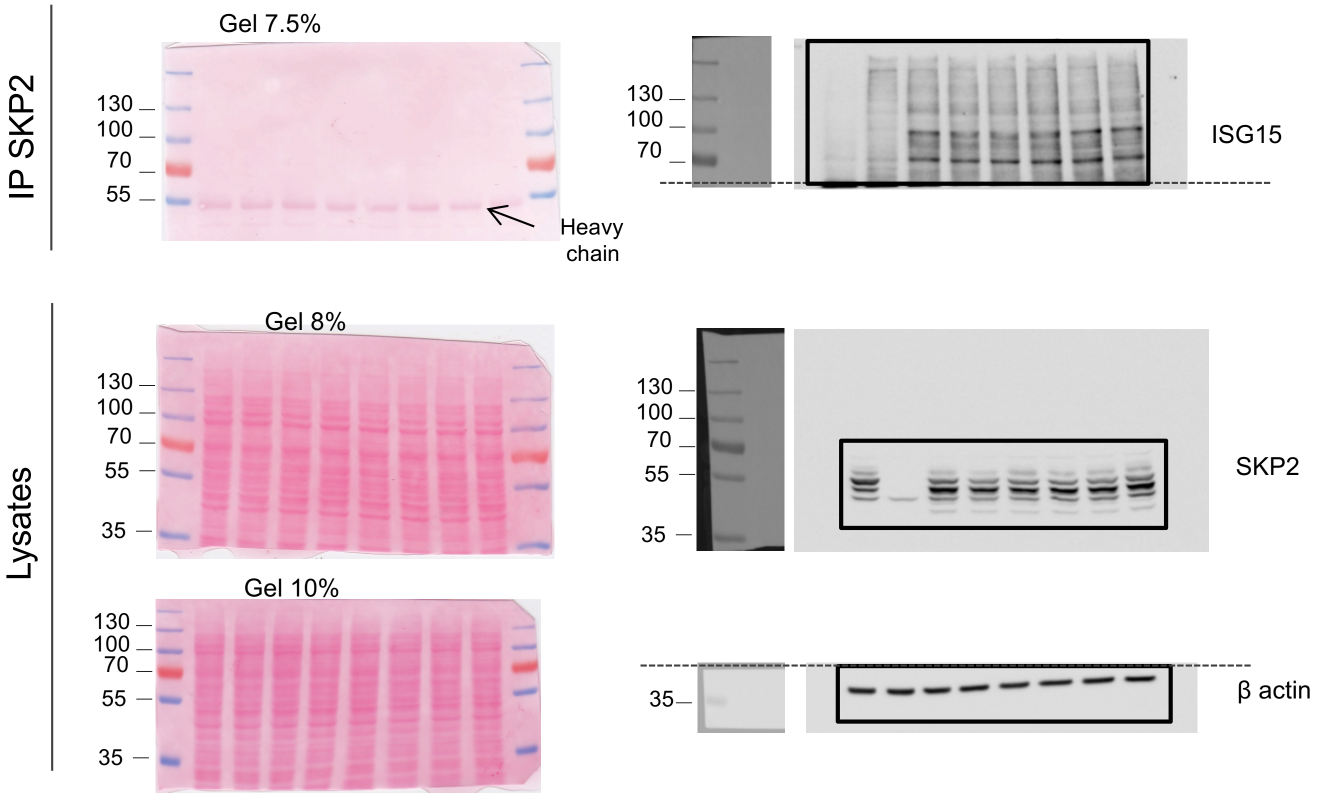

Panel #4

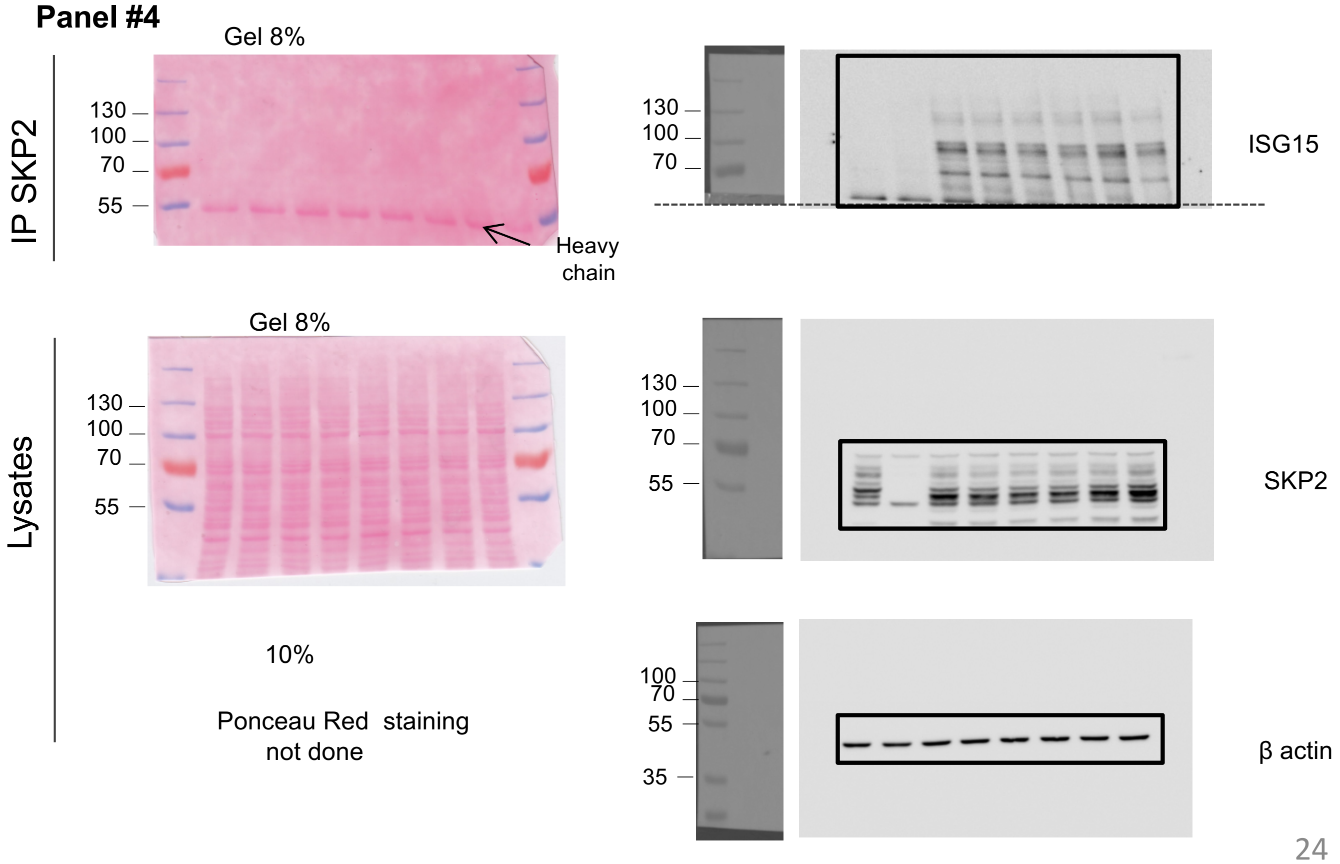

Supplementary Fig. S22

Full-length western blot of Supplementary Fig. S3C (panel 5)

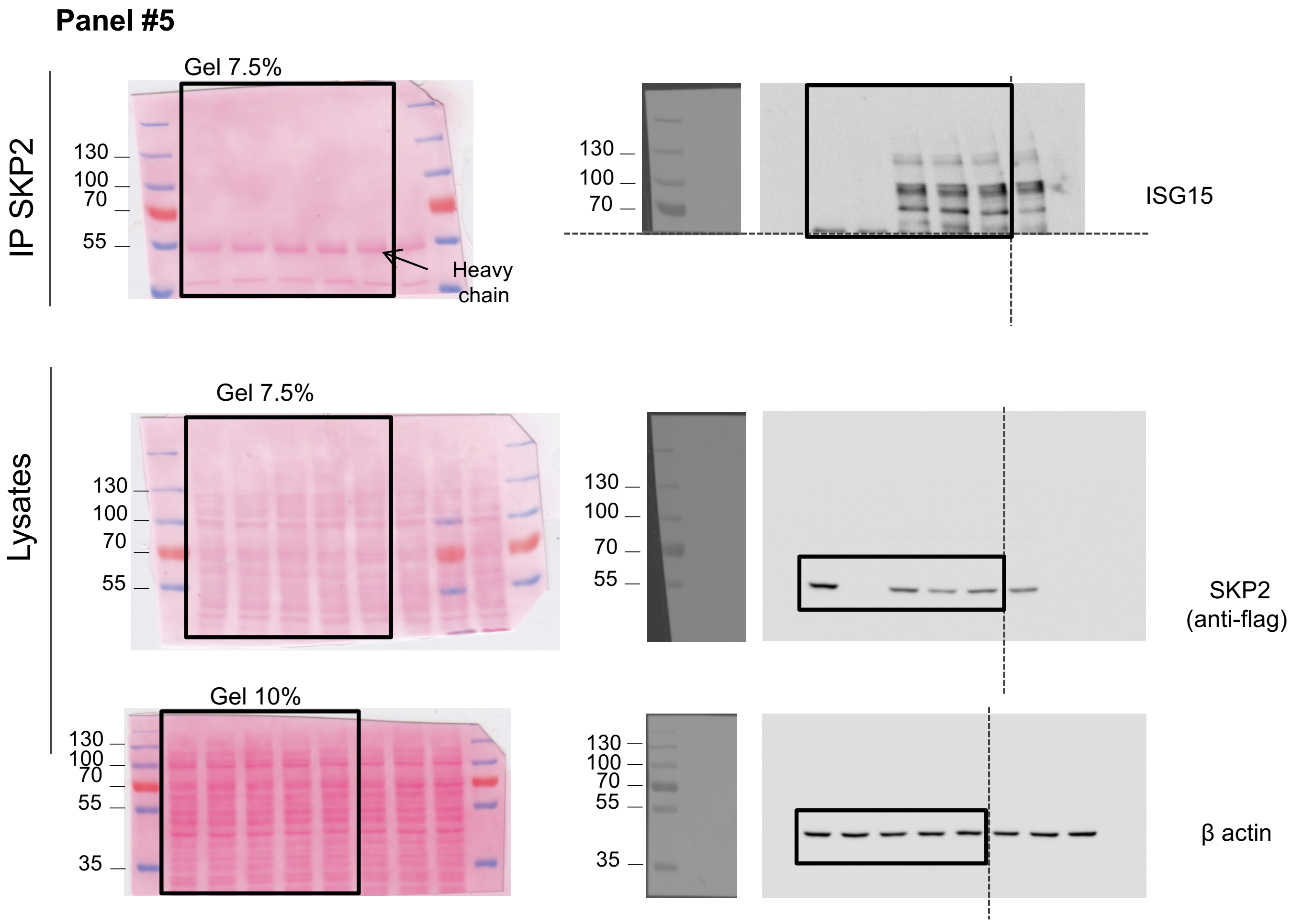

Supplementary Fig. S23
Full-length western blot of Supplementary Fig. S4A

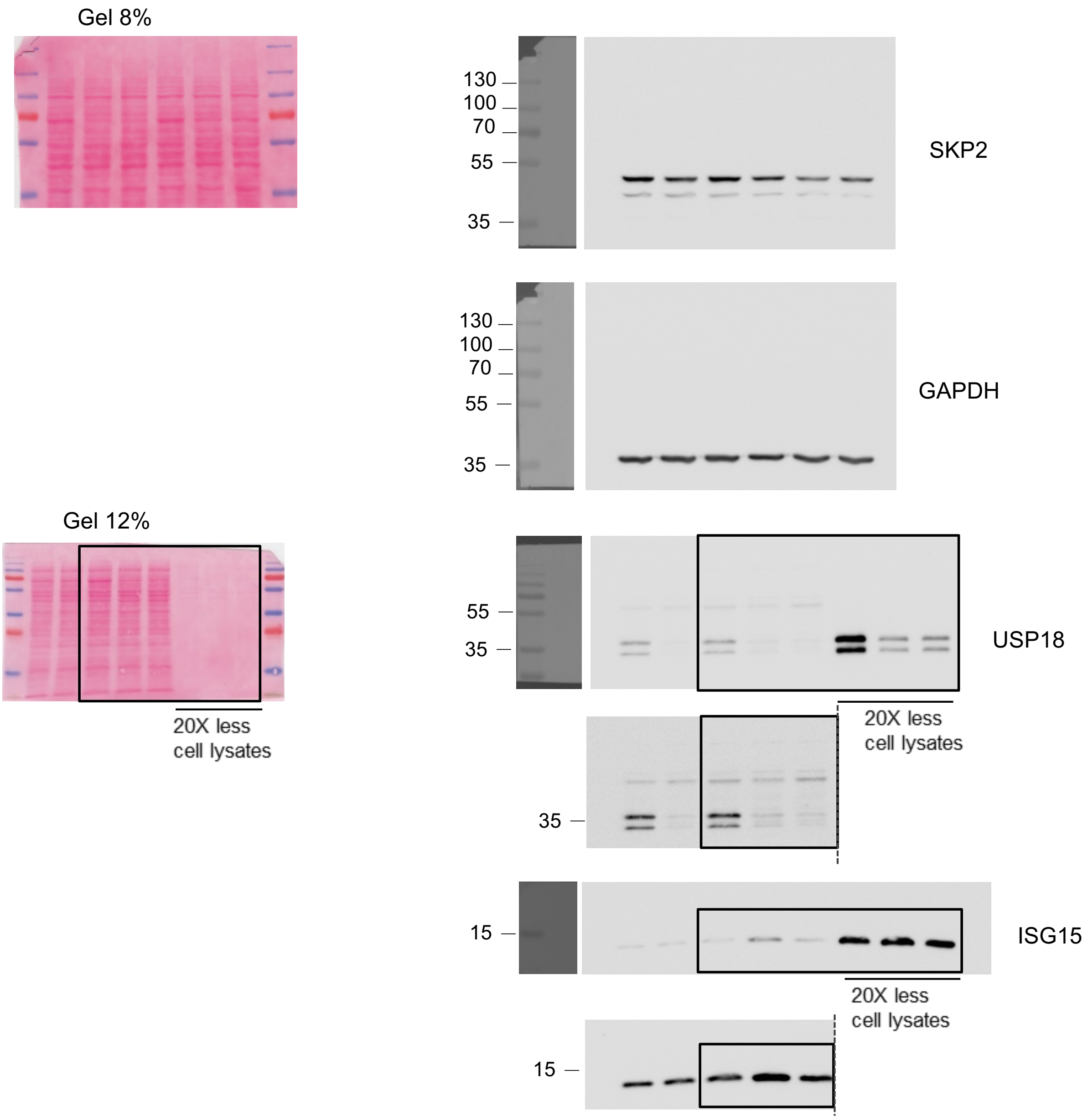

Supplement: Supplementary file 1 — Supplementary information [file 41598_2019_39343_MOESM1_ESM.pdf]
